# High-Value Brown Algae Extracts Using Deep Eutectic Solvents and Microwave-Assisted Extraction

**DOI:** 10.3390/foods14132280

**Published:** 2025-06-27

**Authors:** Meirielly Jesus, Aloia Romaní, Joana Santos, Preciosa Pires, Pablo Del-Río, Fernando Mata, Élia Fernandes, Carla Ramos, Manuela Vaz-Velho

**Affiliations:** 1 CISAS-Center for Research and Development in Agrifood Systems and Sustainability, Instituto Politécnico de Viana do Castelo, Rua da Escola Industrial e Comercial Nun’Alvares 34, 4900-347 Viana do Castelo, Portugaljoana@estg.ipvc.pt (J.S.); ppires@estg.ipvc.pt (P.P.); fernandomata@ipvc.pt (F.M.); eliaf@estg.ipvc.pt (É.F.); 2 Departamento de Enxeñaría Química, Facultade de Ciencias, Universidade de Vigo, 32004 Ourense, Spain; 3 Instituto de Agroecoloxía e Alimentación (IAA), Universidade de Vigo–Campus Auga, 32004 Ourense, Spain; 4 ESTG-IPVC—Escola Superior de Tecnologia e Gestão, Instituto Politécnico de Viana do Castelo, Avenida do Atlântico, 644, 4900-348 Viana do Castelo, Portugal; cramos@estg.ipvc.pt

**Keywords:** antimicrobial properties, *Ascophyllum nodosum*, bioactive compounds, deep eutectic solvents, *Laminaria hyperborea*, microwave extraction, green chemistry, bioresource valorization

## Abstract

Utilizing deep eutectic solvents (DESs) combined with microwave-assisted extraction (MAE) provides a sustainable method for extracting bioactive compounds from the macroalgae *Ascophyllum nodosum* and *Laminaria hyperborea*. Two DES formulations, choline chloride/lactic acid (ChCl/LA) and sodium acetate/lactic acid (AcNa/LA), were evaluated under varying extraction conditions. For *L. hyperborea*, ChCl/LA at 150 °C for 10 min yielded a total phenolic content (TPC) of 15.34 mg GAE/g DW, with antioxidant activities measured by DPPH (34.55 mg TE/g DW) and ABTS (27.06 mg TE/g DW). Extending the extraction to 20 min at 130 °C increased the TPC to 19.12 mg GAE/g DW*. A. nodosum* exhibited higher bioactivity, with the TPC reaching 47.51 mg GAE/g DW under the same conditions. High-performance liquid chromatography (HPLC) identified significant phenolics such as 3,4-dihydroxybenzoic acid (678.05 µg/g DW) and vanillin (6718.5 µg/g DW). Antimicrobial assays revealed strong inhibition (zones > 20 mm) against *Clostridium perfringens,* moderate activity against *Staphylococcus aureus,* and selective activity against *Escherichia coli*. FT-IR confirmed the presence of phenolics, polysaccharides, and lipids. Thermal and structural characterization revealed that *A. nodosum* residue showed an amorphous structure, while *L. hyperborea* retained crystallinity with decomposition profiles indicating potential bioenergy potential. SEM images revealed significant cell wall disruption correlating with extraction efficiency. These results demonstrate DES–MAE as an effective, green strategy for producing high-value algal extracts and valorizing residual biomass for biotechnological applications.

## 1. Introduction

Brown macroalgae have emerged as a rich source of structurally diverse bioactive compounds, attracting growing interest due to their potential applications in the food, cosmetic, pharmaceutical, and nutraceutical industries. Among these, species such as *Laminaria hyperborea* and *Ascophyllum nodosum* are particularly notable for their high content of phenolic compounds, phlorotannins, terpenoids, carotenoids, alginates, and fucoidans. These secondary metabolites have been widely studied for their potent biological activities, including their antitumor, immunomodulatory, antibacterial, anti-inflammatory, and antioxidant properties [1,2,3,4]. *L. hyperborea* and *A. nodosum* were selected for this study not only due to their high bioactive potential reported in the literature but also because they are abundant and readily available in the geographic region where this research was conducted. Their richness in biofunctional phenolics and polysaccharides makes them highly promising candidates for the development of health-promoting functional ingredients in food, pharmaceuticals, and health-promoting products [4,5].

In recent years, substantial research has been dedicated to the development of efficient and sustainable extraction technologies capable of selectively isolating these compounds from complex algal matrices. Advances include the use of environmentally friendly solvents such as deep eutectic solvents (DESs) and ionic liquids, and the implementation of green extraction techniques like microwave-assisted extraction (MAE), ultrasound-assisted extraction (UAE), enzyme-assisted extraction (EAE), and supercritical fluid extraction (SFE). These technologies have shown the potential to enhance extraction yield and selectivity while minimizing environmental impacts and reducing the use of hazardous organic solvents [6,7].

Despite these advances, challenges persist due to the rigid polysaccharide-rich structure of brown algal cell walls and the diverse polarity of target metabolites, which hinder extraction efficiency. Conventional extraction methods such as hydrodistillation, Soxhlet extraction, and solvent extraction are often associated with long processing times, low selectivity, and the use of toxic solvents, compromising both environmental sustainability and product safety [8,9].

Green extraction methods, particularly MAE combined with DESs, have therefore attracted considerable interest as more effective, ecologically responsible alternatives [10,11]. Low toxicity, high biodegradability, and selectivity in the extraction of bioactive compounds are characteristics of DESs, which are created by combining biodegradable components like choline chloride, organic acids, sugars, or amino acids [12,13]. There is a growing trend of using these tactics in algal biorefinery platforms. For instance, Hilali and co-authors [14] suggested a three-step method using DESs to extract fatty acids, chlorophylls, and carotenoids, while Jesus and co-authors [15] combined MAE and UAE with DESs to recover phenolic compounds from *Sargassum muticum*. Lopes and co-authors [11] used MAE for carotenoids in *Himanthalia elongata*, illustrating how hybrid techniques increase extraction while lowering environmental effects. These developments highlighted the critical importance of macroalgae in the marine bioeconomy. They are in line with the circular economy and the United Nations’ Sustainable Development Goals (SDGs), especially SDG 12 (Responsible Consumption and Production) and SDG 13 (Climate Action).

Although previous studies have explored the use of MAE combined with DESs or natural deep eutectic solvents (NADESs) for extracting bioactive compounds from various macroalgae [10,13,14,15], there remains a significant gap regarding the optimization and application of specific DES formulations, such as sodium acetate/lactic acid (AcNa/LA) and choline chloride/lactic acid (ChCl/LA), for the sustainable extraction of phenolic and polysaccharide-rich compounds from *A. nodosum* and *L. hyperborea*. This study hypothesizes that combining MAE with these tailored DES formulations can overcome existing challenges related to extraction efficiency and selectivity imposed by the complex cell wall matrix and metabolite polarity, leading to improved yields and bioactivities. Thus, the novelty of this work lies in the systematic optimization and comprehensive evaluation of these specific MAE-DES combinations on these two abundant brown macroalgae species, filling an important knowledge gap and advancing the development of eco-friendly and scalable extraction methods.

In this context, the current study explores the use of MAE in combination with two DES formulations, AcNa/LA and ChCl/LA, for the environmentally friendly extraction of bioactive compounds from *A. nodosum* and *L. hyperborea*. The specific goals are as follows: (i) characterizing the physicochemical composition of the algal biomass; (ii) optimizing the extraction parameters (temperature, time, and solvent composition); (iii) using HPLC to perform chemical characterization of the extracts; (iv) assessing the antioxidant activity using DPPH, ABTS, and FRAP assays; (v) testing the antimicrobial potential against *Staphylococcus aureus*, *Clostridium perfringens*, and *Escherichia coli*; (vi) identifying functional groups using FT-IR spectroscopy; and (vii) characterizing the residual biomass with the goal of secondary valorization. This work supports the development of sustainable marine bioproducts and the integration of ecologically responsible procedures in valorizing algal biomass by creating a scalable and eco-efficient extraction approach.

## 2. Materials and Methods

### 2.1. Raw Materials and Chemical Composition Analysis

Samples of *L. hyperborea* were collected at Castelo de Neiva (41°3′09″ N 8°4′46″ W), and *A. nodosum* were collected at Praia Norte (41°41′36″ N 8°50′48″ W), Viana do Castelo, Portugal, in April 2023. In the laboratory, the algae were thoroughly washed with fresh tap water to remove salts and impurities. Subsequently, the samples were dried overnight at 45 °C in a convection oven. The dried material was ground to a uniform size using a mill and sieved to particles of 8 mm. The proximate composition of algae was determined using a combination of standardized methods developed by the National Renewable Energy Laboratory (NREL) and AOAC protocols [16,17,18]. The analyses included the evaluation of extractives, moisture content, ash content, crude protein, and fat content. Moisture content was measured by drying the sample in an oven at 103 °C until a constant weight was achieved, according to AOAC 930.04. Ash content was determined by incinerating the sample at 550 °C, as outlined in AOAC 930.05. Crude protein content was calculated as nitrogen × 6.25, employing the Kjeldahl method (AOAC 978.04).

These standardized procedures ensured accurate and reliable compositional profiling of the algae. The Soxhlet method was employed to determine the extractive content, using water and ethanol as solvents [17]. Quantitative acid hydrolysis was performed to evaluate the polysaccharide content [16]. The liquid phase obtained during hydrolysis was analyzed using high-performance liquid chromatography (HPLC) with a refractive index detector and an Aminex HPX-87H column (300 × 7.8 mm, Bio-Rad Laboratories inc., Hercules, CA, USA). The elution process utilized 0.005 M H_2_SO_4_ as the mobile phase at a flow rate of 0.6 mL/min, with the column temperature maintained at 60 °C. The concentrations of the sugars present in the hydrolysate were calculated from standard calibration curves. Following qualitative acid hydrolysis, the insoluble solid residue was recovered and weighed to determine the insoluble fraction via gravimetry.

### 2.2. Microwave-Assisted Extraction

Phenolic compounds were extracted using Teflon vessels in the Ethos X Microwave Extraction System (Milestone Inc., Monroe, CT, USA) under various conditions to optimize yield and antioxidant activity. This study evaluated two DESs, ChCl/LA and AcNa/LA, as alternative solvents in microwave-assisted extractions. The extraction involved a liquid-to-solid ratio of 50 mL solvent (ethanol–water or DES–water mixtures) per 5 g of seaweed. The solvent compositions tested included 50% ethanol–water, 100% water, and DES–water mixtures ranging from 60% to 100%.

**Figure 1:**
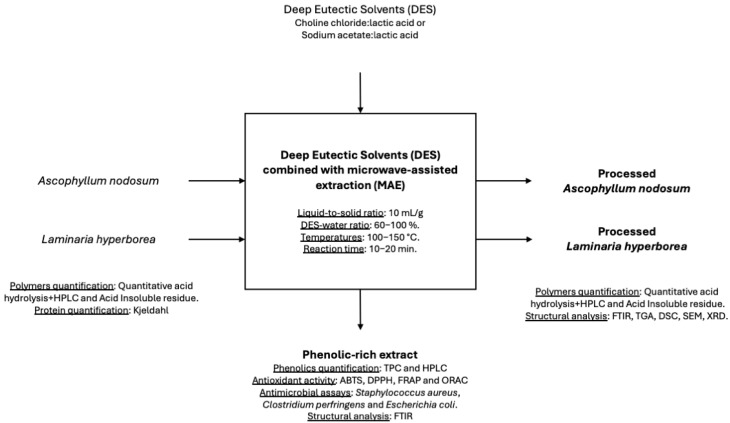
Schematic representation of the experimental workflow, including solvent selection, microwave-assisted extraction, optimization of conditions, analysis of phenolic content and antioxidant activity, and characterization of the solid residue after extraction.

Initial extractions were performed at 100 °C for 10 min. Based on the results of total phenolic content (TPC) and antioxidant activity, further extractions were conducted at higher temperatures (130 °C and 150 °C) and durations (10 and 20 min). The extracts were recovered by filtration and stored at −20 °C until analysis. All experiments were performed in triplicate. A schematic representation of the extraction procedure is provided in Figure 1 to better illustrate the methodology applied in this study.

### 2.3. Phenolic Compound Analysis and Antioxidant Activity

The total phenolic content (TPC) of the brown algae extracts was determined using the Folin–Ciocalteu reagent, adapted for a 96-well microplate format [19]. A reaction mixture containing 10 μL of the sample, 60 μL of sodium carbonate solution (7.5% *w*/*v*), 15 μL of Folin–Ciocalteu reagent, and 200 μL of distilled water was incubated at 60 °C for 6 min. The absorbance was measured at 700 nm using a UV-Vis microplate reader (Varioskan LUX, Thermo Fisher Scientific Inc., Waltham, MA, USA). The TPC was quantified using a gallic acid standard curve (Y = 0.0007x + 0.0671, r = 0.99433), and the results were expressed as milligrams of gallic acid equivalents per gram of dry weight (mg GAE/g DW).

The antioxidant activity of the algae extracts was evaluated using ABTS, DPPH, and FRAP assays, following established protocols [13,20]. These assays assess the ability of the extracts to scavenge free radicals and reduce oxidizing agents, providing a comprehensive profile of antioxidant potential. For the ABTS assay, ABTS^+^ radicals were generated by reacting 7.4 mM ABTS with 2.6 mM potassium persulfate and incubating the mixture in the dark for 16 h. The absorbance was adjusted to 0.700 at 734 nm before the extracts were added. In the DPPH assay, a DPPH radical solution (6 × 10^−5^ M in methanol) was prepared, and the absorbance was measured at 515 nm after 1 h of incubation in the dark. The results for both assays were expressed as Trolox equivalents (mg TE/g DW). The ferric reducing antioxidant power (FRAP) was assessed using a reagent composed of 0.3 M acetate buffer, 10 mM TPTZ, and 40 mM ferric chloride in a 10:1:1 (*v*/*v*/*v*) ratio. The extracts were added to the reagent and incubated at 37 °C for 15 min, followed by absorbance measurement at 593 nm. The results were expressed as ferrous equivalents (g Fe/100 g DW). The phenolic composition of the extracts was determined using HPLC (Agilent 1260 series, Palo Alto, CA, USA) with an AB SCIEX Triple Quad 3500 detector (AB Sciex, Foster City, CA, USA) equipped with an electrospray source of ionization (ESI). The extracts were filtered through a 0.45 µm membrane before injection. Chromatographic separation was performed using a Luna C18 (Phenomenex, Torrance, CA, USA) column (250 × 4.6 mm, 5 µm) at 30 °C. Two mobile phases consisting of 0.1% formic acid in water (A) and 0.1% formic acid in acetonitrile (B) were employed under a gradient elution: 5% B at 0 min, linearly increasing to 50% B over 20 min, followed by re-equilibration. The flow rate was 0.4 mL/min, with an injection volume of 5 µL and a positive/negative source of ionization with turbo V™, with nitrogen as a nebulizer and collision gas. Multiple reaction monitoring (MRM) was employed to obtain the data [19]. Phenolic compounds were identified by comparison with reference standards, including 3,4-dihydroxybenzoic acid, phthalic acid, salicylic acid, 4-hydroxybenzoic acid, and vanillin. Concentrations were determined using calibration curves and expressed in µg/g DW. All analyses were performed in triplicate for accuracy.

### 2.4. Protein Content of Extracts

The protein content of the extracts was determined using the Bradford method, adapted for 96-well microplate format. Briefly, 10 µL of each sample or a bovine serum albumin (BSA) standard (0–1000 µg/mL) was pipetted into the wells of a 96-well microplate. Subsequently, 200 µL of Bradford reagent was added to each well. The plate was incubated at room temperature for 5 min to allow color development. Absorbance was measured at 595 nm using a microplate reader (Varioskan LUX, Thermo Fisher Scientific Inc., Waltham, MA, USA). The results were expressed as BSA equivalents (g BSA/g DW) [21].

### 2.5. Purification of Extracts

To enhance the bioactivity of the crude extracts obtained from *L. hyperborea* and *A. nodosum* using DESs (60% ChCl/LA and 60% AcNa/LA), a purification step was performed. This process aimed to remove DES components and concentrate bioactive compounds in a solvent-compatible fraction for biological assays.

Purification was carried out using a liquid–liquid extraction with ethyl acetate, a solvent known for its high affinity for phenolic compounds and other bioactive molecules, with an extract/solvent ratio of 1:3 (*v*/*v*) at room temperature under stirring for 15 min in two extraction stages [22]. The upper layer containing bioactive compounds was collected, evaporated to dryness, and resuspended in a concentration of 20 µg/mL for further antimicrobial testing. The lower aqueous phase, which contained residual DES and water, was discarded. This approach effectively enriched the bioactive fraction, as ethyl acetate preferentially extracts hydrophobic and moderately polar compounds.

### 2.6. Fourier Transform Infrared (FT-IR) Analysis

The functional groups and bonding arrangements present in the extracts and residue solids were characterized using Fourier transform infrared (FT-IR) spectroscopy. A Nicolet iS20 FT-IR spectrometer, equipped with an attenuated total reflectance (ATR) accessory featuring a diamond composite crystal, was used for the analysis.

The FT-IR spectra were recorded in the frequency range of 400–4000 cm^−1^ with a resolution of 4 cm^−1^, using 30 scans per sample to enhance signal quality [15]. The algal extracts were purified using ethyl acetate and subsequently resuspended in DMSO at a concentration of 250 µg/mL.

### 2.7. Antimicrobial Activity

The antimicrobial activity of the extracts was evaluated using the disk diffusion method, following CLSI guidelines [23]. The extracts with the highest antioxidant activity were selected for testing against *Clostridium perfringens* (ATCC 13124), *Escherichia coli* (ATCC 25922), and *Staphylococcus aureus* (ATCC 25923). Bacterial strains were cultured on Columbia Agar supplemented with 5% sheep blood (COS, Biomérieux, Craponne, France). The cultures were adjusted to a turbidity equivalent to the 0.5 McFarland standard and uniformly spread onto Mueller–Hinton Agar (MHA, Oxoid Ltd., Basingstoke, UK) using sterile cotton swabs.

For the disk diffusion assay, sterile 6 mm paper disks (Oxoid Ltd., Basingstoke, UK) were impregnated with 10 µL of each test extract. The agar plates were divided into eight equal sections, and the disks were placed on the surface, including the following controls: negative control: DES solutions (60% ChCl/LA or 60% AcNa/LA); positive control: commercial sodium hypochlorite solution (Neoblanc, Fater SpA, Spoltore, Italy). After drying for 15 min at room temperature, the plates were incubated at 37 °C ± 1 °C for 22 ± 2 h. The diameter of the inhibition zones was measured in millimeters using ImageJ software version i.46r, National Institutes of Health, Bethesda, MD, USA. Each measurement was performed in triplicate, and the results were expressed as the mean diameter of the inhibition halos. The bacterial strains were chosen for their relevance as foodborne pathogens and spoilage organisms, representing both Gram-positive and Gram-negative bacteria.

### 2.8. Structural Analysis

The thermal stability and behavior of the solid residues obtained after bioactive compound extraction were evaluated using differential scanning calorimetry (DSC) and thermal gravimetric analysis (TGA) [24]. The analysis was carried out with a DSC 6000 analyzer (PerkinElmer, Waltham, MA, USA) to determine the thermal transitions and degradation patterns of the samples. The temperature range for the analysis was set between 25 °C and 600 °C, with a controlled heating rate of 10 °C/min under a nitrogen atmosphere to minimize oxidation. This method provided critical insights into the thermal properties and decomposition behavior of the extracted residues, aiding in the assessment of their potential for thermal stability and subsequent applications.

The microstructure of the solid residues obtained after the extraction of bioactive compounds from algae was analyzed using a field-emission scanning electron microscope (FEG-SEM) (Jeol JSM-7001F, Tokyo, Japan). Micrographs were captured at 400x magnification with an accelerating voltage of 5 kV. To minimize charge buildup during imaging, the samples were pre-coated with a thin layer of Au-Pd alloy before observation. X-ray diffraction (XRD) was conducted to characterize the crystalline and amorphous phases present in the solid residues remaining after bioactive compound extraction. A D8 Advance DaVinci diffractometer (Bruker AXS, Karlsruhe, Germany) was used, equipped with Ni-filtered Cu-Kα radiation (λ = 0.15418 nm) generated at 40 kV and 30 mA and a Lynxeye 1-D linear detector [24].

The diffraction patterns were recorded over a 2θ range of 5 °C to 60 °C, with a step size of 0.02° and a counting time of 0.5 s per step. During data acquisition, the samples were rotated at 15 rpm to ensure uniform exposure. Rietveld refinement was performed using TOPAS 5.0 (Bruker AXS, Karlsruhe, Germany) with the fundamental parameter approach for precise phase identification and quantification. Zinc oxide was used as an internal standard to enable the quantification of the amorphous content in the residues. This analysis provided detailed insights into the structural composition of the residues, aiding in the evaluation of changes induced by the extraction process.

### 2.9. Statistical Analysis

For the differentiation of the chemical composition between algae, data were analyzed using an independent-sample Student’s t-test. As there were only 3 biological replicates per algae species *(n* = 3), we extended the data set by performing bootstrapping stratified by species to generate 100 samples. For the remaining analyses, data were obtained from triplicate extractions (*n* = 3) and analyzed using full factorial type III ANOVAs. The post hoc test used was the least significant difference (LSD) test, as no more than three levels were found for each factor. The prerequisites of the ANOVAs were checked with the Levine’s test and the Shapiro–Wilk test for the homogeneity of variances and normal distribution of the residuals, respectively. The analysis was performed using the statistical package IBM Corp^®^ SPSS^®^ 29.0.2.0 (20), Armonk, NY, USA. Statistical significance was set at *p*  <  0.05.

## 3. Results and Discussion

### 3.1. Chemical Composition

The chemical composition of the studied algae provides significant insights into their potential as a source of valuable bioactive compounds. The results indicate a notable presence of polysaccharides, proteins, and other organic materials, which are essential for applications in food, pharmaceuticals, and biotechnology [25,26].

The presence of glucans (6.11 g/100 g) and rhamnan (9.35 g/100 g) in *L. hyperborea* is particularly noteworthy (Table 1). Glucans are recognized for their health benefits, including immunomodulatory effects and prebiotic properties that can stimulate beneficial gut bacteria [27,28], while rhamnose contributes to structural cell wall integrity and displays antioxidant and anti-inflammatory effects [29,30]. These findings are consistent with prior studies on green algae polysaccharides.

Although no statistically significant differences were observed, *L. hyperborea* showed a numerically higher glucan content (6.11 g/100 g) compared to *A. nodosum* (2.48 g/100 g), whereas *A. nodosum* presented a higher fucoidan concentration (3.20 g/100 g vs. 1.46 g/100 g). Fucoidan, a sulfated polysaccharide, is known for its diverse bioactivities, has demonstrated diverse bioactivities, including anticoagulant, antitumor, antithrombotic, antinflammatory, antiangiogenic, immunity-boosting, and antiviral effects [29,30,31]. Although present in smaller amounts, xylan, galactan, and mannan (1.46 g/100 g in *L. hyperborea* vs. 1.20 g/100 g in *A. nodosum*) enhance the polysaccharide profile of these macroalgae, contributing to their functional properties. Mannans, which share structural similarities with the mannuronic acid blocks of alginates, contribute to increased viscosity and improved stability in polymeric solutions [32]. The presence of these complementary polysaccharides further supports the potential of *L. hyperborea* and *A. nodosum* as rich sources of multifunctional ingredients for sustainable biotechnological applications [33].

The protein content is higher in *L. hyperborea* in comparison to *A. nodosum*. Nevertheless, both algae contain a considerable protein content. Algal proteins are increasingly recognized as sustainable sources of essential nutrients. They provide essential amino acids and bioactive peptides with therapeutic properties, such as antihypertensive and anticancer effects [34,35]. This protein content, considering the growing demand for alternative proteins, positions these algae as a promising source.

The high levels of water extractives and ethanol indicate the abundance of soluble compounds with bioactive potential, such as phenolics, flavonoids, and other antioxidant compounds [28]. In particular, water-soluble compounds may include polysaccharides like alginates, widely used in food preservation, cosmetics, and pharmaceuticals [36]. These results suggest that algae could be a valuable raw material in biorefinery processes for extracting high-value compounds.

The ash content of the algae is essential for various physiological functions and contributes to the nutritional profile [37]. It reflects the ability of algae to absorb minerals from seawater, making them a sustainable alternative to traditional mineral sources. Elements such as calcium, magnesium, potassium, iron, and iodine are common in algae and vital for human health [28,38].

These results align with previous studies on *L. hyperborea* [39,40], which emphasized the high mineral content in brown algae. The chemical composition of the studied algae, rich in bioactive polysaccharides, proteins, and extractives, highlights their potential for the development of innovative and sustainable products. These distinctions emphasize the diverse applications of each species, from mineral-rich supplements to sources of bioactive polysaccharides. These products could meet the growing demand for natural bioactive compounds in the food, cosmetic, and pharmaceutical sectors. The sustainable extraction of these compounds is crucial, requiring the development of efficient and environmentally friendly methods to maximize the utilization of these marine resources without compromising environmental integrity.

### 3.2. Preliminary Extraction

The preliminary extraction ANOVA tests revealed significant differences in the extraction efficiency of bioactive compounds, including TPC (F = 149.5, 12 df, *p* < 0.001) and antioxidant activity DPPH (F = 170581, 12 df, *p* < 0.001) and ABTS (F = 227, 12 df, *p* < 0.001), depending on the type and concentration of solvent used, and the algae species. The results are summarized in Table A1 (factors), Table A2 (first-order interactions), and Table 2 (second-order interactions). The choice of solvent is a crucial factor in maximising the extraction of bioactive compounds, as demonstrated by Jesus et al. [19].

The preliminary extraction ANOVA results reveal the significant impact of both the solvent type and concentration, as well as the algal species, on the efficiency of bioactive compound extraction. Notably, the extraction of total phenolic content (TPC) and the antioxidant activities measured by DPPH and ABTS assays exhibited highly significant variations (*p* < 0.001). These findings align with the existing literature emphasizing the pivotal role of extraction parameters in maximizing the yield and activity of bioactive compounds from algae [41,42].

The extraction efficiency varied with the solvent concentration, revealing that a lower concentration (60%) was more effective in extracting phenolic compounds, as evidenced by the higher TPC values (5.92 mg GAE/g DW). These results are consistent with studies suggesting that moderate–polarity solvents are ideal for phenolic extraction due to their compatibility with the solubility of these compounds [43]. Conversely, higher solvent concentrations (100%) favored the extraction of compounds measured by the ABTS assay, reflecting the role of solvent polarity in selectively isolating specific antioxidant components [44].

The algal species *L. hyperborea* showed a superior TPC (6.28 mg GAE/g DW) compared to *A. nodosum* (4.31 mg GAE/g DW), supporting previous findings that highlight species-specific differences in bioactive compound profiles due to variations in metabolic pathways and environmental adaptations [45]. Despite this, the DPPH activity exhibited minor variations between species, suggesting that phenolics are not the sole contributors to radical scavenging capacity.

The interaction between the solvent type and algae species significantly influenced extraction outcomes. For example, the combination of *A. nodosum* with ChCl/AL at 60% solvent concentration resulted in the highest TPC (10.28 mg GAE/g DW). Such findings emphasize the importance of tailoring solvent systems to specific algal matrices for optimal extraction efficiency [46].

Antioxidant activities measured via DPPH and ABTS assays were consistently significant across factors. The DPPH activity was relatively stable, with minor fluctuations attributed to solvent and species interactions, while ABTS activity showed greater sensitivity to the solvent concentration. These results reinforce the necessity for optimized extraction protocols tailored to the specific characteristics of the target bioactive compounds and the algal source. Such optimization is crucial for applications in food, cosmetics, and pharmaceuticals, where the efficacy and purity of extracted bioactive compounds are critical [47].

Overall, the preliminary extraction results underscore the critical influence of solvent composition and algal species on the recovery of bioactive compounds. Moderate-polarity solvents, particularly at 60% concentration, enhanced phenolic extraction, while higher concentrations improved the yield of certain antioxidant components. *L. hyperborea* generally yielded a higher TPC, reflecting species-specific potential. These findings highlight the necessity of tailoring extraction conditions, especially the solvent type and concentration, to each algal matrix for optimal efficiency. Such strategic optimization supports the development of effective and sustainable extraction protocols for bioactive compound recovery in industrial applications.

### 3.3. Phenolic Compound Analysis and Antioxidant Activity

In *A. nodosum*, the lowest concentration of ChCl/LA solvent (60%) resulted in 10.28 mg eq. GAE/g, which was superior to the other concentrations. Conversely, in *L. hyperborea*, phenolic compound extraction was more efficient with the 100% AcNa/LA solvent, yielding 6.18 mg eq. GAE/g (Table 2). These results align with the literature suggesting the effectiveness of ionic solvent combinations, such as choline chloride (ChCl) with sodium acetate (AcNa), in extracting phenolic compounds from marine algae due to their ability to break down the algae cell walls and extract both water- and lipid-soluble compounds [48]. The recent literature also suggests that combining aqueous solvents with high salt concentrations can promote more efficient extraction of phenolic compounds, due to the increased solubility of various bioactive compounds [49].

Regarding antioxidant activity, the 60% ChCl/LA and 60% AcNa/LA concentrations stood out in both algae. The DPPH values showed good performance, especially in *L. hyperborea*, with 14.48 mg eq. Trolox/g (DPPH) for the 60% AcNa/LA solvent. These results are consistent with trends observed in other studies, indicating that solvents with high concentrations of salts and mixtures of solvents like water and ethanol can significantly improve the antioxidant activity of algae extracts [50]. Antioxidant effects often correlate with the presence of phenolic compounds and polyphenols, which have a significant ability to neutralize free radicals [20]. In this context, the use of AcNa/LA at higher concentrations has been highlighted as an efficient solvent for extracting antioxidant compounds from brown algae due to its high ability to dissolve these compounds [51].

While ethanol–water-based solvents, such as the 50% EtOH–water solution, showed some efficiency, they did not surpass ChCl/LA and AcNa/LA mixtures in extracting phenolic compounds and antioxidant activity. This is in line with studies showing that organic solvents combined with acids or salts can enhance the solubility of bioactive compounds, in contrast to purely aqueous solvents [1].

On the other hand, a recent study emphasized the importance of optimizing the solvent concentration to improve the recovery of bioactive compounds without compromising antioxidant activity, showing that too high concentrations of certain solvents can reduce efficiency due to solution saturation [6]. The solvent type and concentration significantly influenced phenolic extraction and antioxidant activity. *A. nodosum* performed best with 60% ChCl/LA, while *L. hyperborea* showed better results with 100% AcNa/LA. Antioxidant activity correlated with phenolic content, highlighting the importance of tailored solvent systems for efficient bioactive compound recovery.

### 3.4. Optimizing Extraction Conditions for Enhanced Bioactive Compound Recovery

The preliminary extraction tests provided critical insights into the solvent efficiency and informed the optimization process. These preliminary results revealed the importance of the solvent type and concentration in maximizing the extraction of bioactive compounds such as the TPC and antioxidant compounds, measured by DPPH and ABTS assays. The optimization was undertaken to evaluate the impact of temperature and extraction time, using the most effective solvent concentration identified in the initial phase (60% ChCl/LA and 60% AcNa/LA). These solvents demonstrated a superior capacity to extract TPC and antioxidants, particularly at temperatures of 100 °C, 130 °C, and 150 °C with 10 to 20 min extraction times. By fine-tuning these parameters, the study identified conditions that maximized the recovery of bioactive compounds from the algae.

The optimized extraction ANOVA tests revealed significant differences in the extraction efficiency of bioactive compounds, including TPC (F = 482.1, 16 df, *p* < 0.001), DPPH (F = 728.6, 16 df, *p* < 0.001) and ABTS (F = 349.4, 16 df, *p* < 0.001) antioxidant activity, FRAP (F = 894.1, 16 df, *p* < 0.001), and proteins (F = 1006.7, 16 df, *p* < 0.001), depending on the type of solvent, time, temperature, and the algae species. The results are summarized in Table A3 (factors), Table A4 (first-order interactions), Table A5 (second-order interactions), and Table 3 (third-order interactions).

The analyses of the simple factors show that the extraction is more effective with 20 min than with 10 min, in relation to the phenols (TPC), and antioxidants using ABTS and FRAP. In relation to the temperature, the extraction is also more effective for phenols (TPC) and antioxidants using ABTS at a higher temperature (150 °C). The solvent ChCl/AL is more effective for the extraction of phenols (TPC) and protein. The solvent AcNa/AL is more effective for antioxidant extraction while using the ABTS and FRAP techniques. Finally, in relation to the different algae, *A. nodosum* proves higher yields in all the extracts: TPC, antioxidants (with DPPH, ABTS, and FRAP), and protein.

While considering the more effective combination of solvents, *A. Nodosum* produces higher yields in phenols (TPC) and protein if it is extracted with ChCl/AL, while AcNa/AL is more effective with this algae for antioxidants using any of the techniques. The extractions from *L. hyperborea* phenols with ChCl/AL produce higher yields, while in relation to the antioxidants, the yields depend on the technique. The protein extraction is higher when using ACNa/AL.

While considering the combination of algae and time, generally, the length of the process is associated with higher yields. While considering the combination of algae and temperature, the same was observed with higher temperatures associated with higher yields, as discussed before. The combination of solvent and time, however, produces mixed results, with the solvent AcNa/LA producing higher yields of antioxidants using the ABTS technique. The combinations of solvent and temperature produce higher yields at higher temperatures, as mentioned before. Finally, concerning the combinations between time and temperature, a higher temperature (150 °C) combined with a higher time of exposure is not associated with higher yields for phenols (TPC) and antioxidants using the FRAP technique.

Advancing into the triple interactions, and considering algae, solvent, and time, higher yields for phenols (TPC) and for protein in *A. Nodosum* were obtained with ChCl/LA for 20 min. The same results were obtained for *L. hyperborea* but for 10 min only. In the combination of algae, solvent, and temperature, higher yields are observed for *A. Nodosum* and *L. hyperborea* in phenols (TPC) with ChCl/LA at higher temperature (150 °C). However, higher yields of protein were observed for *A. Nodosum* and ChCl/LA and for *L. hyperborea* and AcNa/LA at lower temperatures (130 °C).

Finally, the overall results indicate that a higher yield of phenols from *A. Nodosum* was achieved using ChCl/LA as a solvent at 130 °C for 20 min. 

The optimized extraction confirmed that both the solvent choice and process parameters (temperature and time) significantly affect the recovery of bioactive compounds. *A. nodosum* yielded higher levels of phenols and proteins with ChCl/LA, while AcNa/LA was more effective for antioxidant extraction. *L. hyperborea* showed better antioxidant performance with ORAC. Overall, higher temperatures (130–150 °C) and longer extraction times (20 min) enhanced yields, although excessive conditions did not always improve results. These findings underscore the need for precise parameter tuning to maximize extraction efficiency.

#### 3.4.1. Total Phenolic Compounds

The extraction of TPC from *L. hyperborea* and *A. nodosum* was significantly influenced by the solvent composition, temperature, and extraction time. The highest TPC concentrations were obtained using 60% ChCl/LA and 60% AcNa/LA solvents under optimized extraction conditions. Specifically, at 150 °C for 10 min, *L. hyperborea* achieved 19.12 mg GAE/g DW (ChCl/LA), and *A. nodosum* reached 47.51 mg GAE/g DW (ChCl/LA). These findings indicate the superior ability of these solvents to extract phenolic compounds, reflecting their potential in bioactive compound recovery from brown seaweeds.

Further optimization studies confirmed that 60% DES (ChCl/LA) at 130 °C for 20 min provided the best yield for TPC, with *L. hyperborea* reaching 15.34 mg GAE/g and *A. nodosum* yielding 50.62 mg GAE/g. This result aligns with previous studies suggesting that choline chloride-based deep eutectic solvents (DESs) are highly efficient in extracting phenolic compounds from seaweed due to their polarity and ability to disrupt the rigid cell walls of algae [48]. The extraction time and temperature have a direct effect on the release of bioactive molecules, with longer extraction times and moderate temperatures often improving yield without leading to degradation. The optimized extraction of TPC demonstrated that the solvent type, temperature, and time critically affect phenolic yield from brown algae. ChCl/LA at 60% was the most effective solvent, particularly at 130–150 °C. *A. nodosum* consistently showed higher TPC values than *L. hyperborea*, confirming species-specific extraction potential. These findings reinforce the efficiency of DESs and highlight the importance of fine-tuning operational conditions to maximize phenolic recovery from seaweed biomass.

#### 3.4.2. Antioxidant Activity

The antioxidant activities of the extracts, measured by DPPH, ABTS, and FRAP assays, were strongly influenced by both the extraction solvent and conditions. *L. hyperborea* exhibited superior antioxidant activity compared to *A. nodosum*, with the highest DPPH and ABTS values observed at 60% DES (ChCl/LA) and 130 °C for 20 min. In particular, *L. hyperborea* showed DPPH values of 35.13 mg TE/g DW and ABTS values of 28.44 mg TE/g DW, while *A. nodosum* demonstrated lower activity, with DPPH at 48.37 mg TE/g DW and ABTS at 83.64 mg TE/g DW. These findings agree with earlier reports which highlighted that *L. hyperborea* possesses a higher concentration of antioxidants, including phenolics and flavonoids, which are known to contribute significantly to its antioxidant capacity [52]. The optimization of extraction parameters further emphasized that higher temperatures (130 °C) and longer extraction times (20 min) are favorable for the enhanced release of antioxidant compounds, especially when using DES-based solvents.

In contrast, *A. nodosum* exhibited higher antioxidant activity across both assays. This is likely due to a higher concentration of antioxidant compounds, which may limit the antioxidant potential of the extract. However, the higher yield of antioxidants obtained from *L. hyperborea* suggests that the species may be a promising candidate for bioactive compound extraction when considering antioxidant activity.

The FRAP assay demonstrated the reducing power of the extracts, with *L. hyperborea* generally exhibiting lower FRAP values compared to *A. nodosum*. For example, the 10 min extraction at 150 °C with ChCl/LA resulted in a FRAP value of 85.04 mg eq. Fe(II)/g DW for *A. nodosum*, while *L. hyperborea* yielded lower values, such as 52.86 mg eq. Fe(II)/g DW. These differences underline the more potent antioxidant power of *A. nodosum*, which is rich in phenolic compounds and other reducing agents [4]. 

The antioxidant activity of the extracts was significantly influenced by the solvent, extraction time, and temperature. *L. hyperborea* showed higher activity in DPPH and ABTS assays, while *A. nodosum* exhibited stronger reducing power in the FRAP assay. These findings highlight the need to tailor extraction conditions based on the specific antioxidant properties desired.

#### 3.4.3. Protein Content

Protein content, measured by the Bradford assay, also showed variations between the two species. *A. nodosum* exhibited higher protein content (18.93 mg eq. albumin/g DW) than *L. hyperborea* (16.80 eq. albumin/g DW). This suggests that *A. nodosum* could be an interesting source for protein extraction and may hold potential for applications in the food and nutraceutical industries [49].

#### 3.4.4. Efficiency of Bioactive Compound Extraction Using Microwave-Assisted Extraction and DESs

The extraction of phenolic compounds was promoted under the optimized solvent concentration and using two different combinations of time and temperature (120 °C for 20 min, 150 °C for 10 min). The optimized extraction ANOVA tests revealed significant differences in the extraction efficiency of 3,4-dihydroxybenzoic acid (F = 1639.9, 8 df, *p* < 0.001), phthalic acid (F = 93.5, 8 df, *p* < 0.001), salicylic acid (F = 59.3, 8 df, *p* < 0.001), 4-hydroxybenzoic acid (F = 1420.6, 8 df, *p* < 0.001), and vanillin (F = 2285.2, 8 df, *p* < 0.001), depending on the type of solvent, the time/temperature combination, and the algae species. The results are summarized in Table A6 (factors), Table A7 (first-order interactions), and Table 4 (second-order interactions).

### 3.5. Bioactive Compound Extraction

#### 3.5.1. Bioactive Compound Extraction in *Ascophyllum nodosum*

The results for *A. nodosum* indicate that both extraction methods, particularly DES-based solvents, were highly effective in extracting bioactive compounds. The highest yields for most of the compounds were obtained when using the 150 °C for 10 min extraction condition, especially with the AcNa/LA solvent. For example, the concentration of vanillin reached an impressive 3981 mg/g, demonstrating the potential of the AcNa/LA solvent at higher temperatures for the extraction of this bioactive compound.

Among the other compounds, DHB (678.1 mg/g) was also present in notable concentrations, with higher yields observed at 150 °C for 10 min. These findings are consistent with previous studies that highlight the potential of DES-based extractions, especially at elevated temperatures, to extract bioactive compounds from seaweeds efficiently [48]. The higher temperature appears to improve the solubility of these compounds and enhance the release from the algal matrix, making AcNa/LA a particularly potent solvent for the extraction of bioactive compounds in *A. nodosum*.

In contrast, when using ChCl/LA at 130 °C for 20 min, lower concentrations of bioactive compounds were observed, especially for vanillin (84.25 mg/g), suggesting that this condition may not be optimal for extracting this compound, despite it being effective for other compounds such as phthalic acid.

#### 3.5.2. Bioactive Compound Extraction in *Laminaria hyperborea*

*L. hyperborea* showed different trends compared to *A. nodosum*, with distinct solvent systems yielding higher concentrations of certain bioactive compounds. For instance, AcNa/LA at 150 °C for 10 min resulted in the highest extraction of vanillin (6718.5 mg/g) and phthalic acid (46.72 mg/g). The increased yield of vanillin under these conditions suggests that *L. hyperborea* is a promising source for vanillin extraction, with AcNa/LA proving to be the most efficient solvent for this compound.

At 130 °C for 20 min, ChCl/LA also yielded significant concentrations of phthalic acid (126.43 mg/g), highlighting the importance of this solvent for extracting certain phenolic compounds in *L. hyperborea*. However, the concentrations of salicylic acid were negligible or zero in many of the extracts, particularly under the ChCl/LA conditions. This suggests that salicylic acid is either less abundant in *L. hyperborea* or less efficiently extracted under the tested conditions.

The data clearly demonstrate that extraction time and temperature are crucial factors for the yield of bioactive compounds. Higher temperatures (150 °C) and shorter extraction times (10 min) generally resulted in higher concentrations of compounds like vanillin, phthalic acid, and 3.4-dihydroxybenzoic acid, particularly when using AcNa/LA as a solvent. These findings align with the existing literature, which has shown that microwave-assisted extraction at elevated temperatures can enhance the diffusion of bioactive molecules from the seaweed matrix and increase the solubility of certain compounds, leading to better yields [19,53].

Conversely, longer extraction times (20 min) at lower temperatures (130 °C) did not always lead to the highest yields, suggesting a threshold beyond which increasing extraction time may not provide additional benefits or may even result in the degradation of sensitive compounds.

#### 3.5.3. Functional Group Characterization by FT-IR Analysis

FT-IR spectroscopy proved instrumental in identifying functional groups in the algae extracts, specifically phenolic compounds, polysaccharides, and lipids. These bioactive compounds are crucial for the observed biological activities, including antioxidant, antimicrobial, and anti-inflammatory properties [54,55].

**Figure 2:**
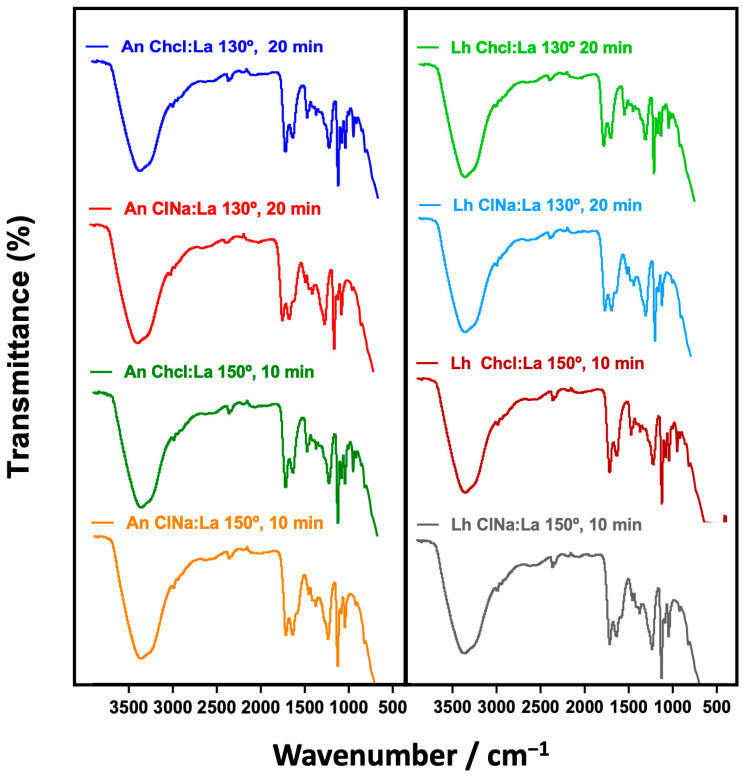
Functional groups identified in extracts of *Laminaria hyperborea* and *Ascophyllum nodosum* obtained using 60% ChCl/LA and 60% AcNa/LA solvents under optimized extraction conditions (130 °C, 20 min; 150 °C, 10 min).

The analysis was conducted on extracts obtained under optimized conditions (130 °C, 20 min) using 60% ChCl/LA and 60% AcNa/LA solvents (Figure 2). The spectra confirmed the presence of bioactive molecules, supporting their potential role in biological activities as previously reported in studies on marine-derived compounds [56]. These results emphasize the importance of FT-IR in elucidating the chemical profiles of marine algae extracts, providing a basis for their application in the food, pharmaceutical, and cosmetic industries [57]. The obtained spectra revealed distinct peaks corresponding to various chemical groups, such as phenolic compounds, carbohydrates, proteins, and other bioactive constituents, providing valuable insights into the molecular composition of the extracts.

The FT-IR analysis of the four crude extracts (*L. hyperborea* and *A. nodosum* extracted with ChCl/AL and AcNa/AL) revealed consistent spectral profiles across all samples, as shown in Figure 1. Key absorption bands were identified, corresponding to phenolic compounds, carbohydrates, and lipids, highlighting the chemical complexity of the extracts. Since the extraction involved DMSO solvents, bands were detected in all spectra. These include a strong band at 1040 cm^−1^, corresponding to SO stretching vibrations, and a broad band at 3260 cm^−1^, associated with O-H stretching [55,58].

Additionally, bands at 2977 cm^−1^ and 2924 cm^−1^ represent C-H stretching vibrations of CH_2_ and CH_3_ groups, commonly found in organic molecules. The FT-IR spectra displayed a band in the region of 1615–1580 cm^−1^, indicating C–H and aromatic C=C–C stretching, confirming the presence of phenolic compounds. Phenolic rings generate characteristic vibrations due to O-H stretching (1242 cm^−1^) and C=O stretching, highlighting the richness of these extracts in polyphenolic compounds [58]. Another significant band at 1067 cm^−1^ corresponds to C–O stretching vibrations, often associated with carbohydrates and alcohols [57]. Additionally, the 1242 cm^−1^ band related to O-H bending vibrations is consistent with findings in seaweed extracts rich in polysaccharides [56]. Bands at 1391 cm^−1^ are indicative of C-H bending vibrations, while 877 cm^−1^ corresponds to C–C stretching vibrations. These signals suggest the presence of saturated hydrocarbons and aliphatic chains, likely originating from lipids present in the algal matrix. The detection of phenolic functional groups, including O-H and C=O, confirms the presence of key bioactive compounds responsible for the antioxidant and antimicrobial properties observed in the extracts. These compounds are known to scavenge free radicals, disrupt microbial cell membranes, and chelate metal ions [55].

Furthermore, the presence of polysaccharides and other carbohydrate-related groups underscores the potential of these extracts for applications beyond antioxidant activity, such as in immunomodulatory and anti-inflammatory therapies [54]. These findings suggest that the extracts from *L. hyperborea* and *A. nodosum* contain a variety of bioactive molecules that could be exploited in various industrial and pharmaceutical applications. Further studies should focus on correlating these functional groups with specific bioactivities and exploring their potential synergies in pharmaceutical and nutraceutical development.

FT-IR spectroscopy identified key bioactive compounds in *L. hyperborea* and *A. nodosum* extracts, phenolics, polysaccharides, and lipids, responsible for antioxidant, antimicrobial, and anti-inflammatory activities. The spectra confirmed these functional groups across all samples, highlighting the extracts’ potential for food, pharmaceutical, and cosmetic applications. Future studies should explore the correlation between these compounds and their biological effects to support functional product development.

#### 3.5.4. Antimicrobial Activity of *Laminaria hyperborea* and *Ascophyllum nodosum* Extracts

**Figure 3:**
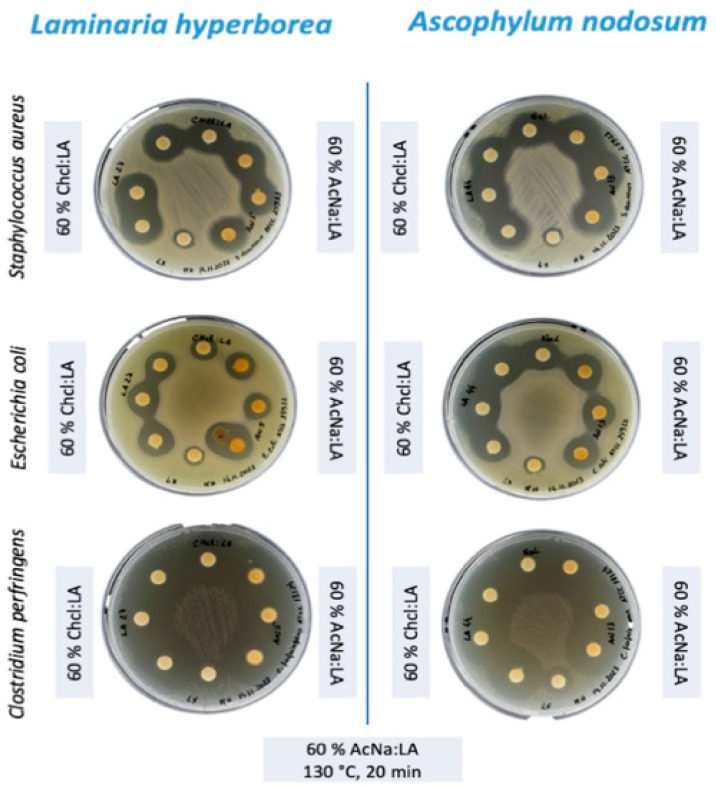
Antimicrobial activity of algal extracts against *Escherichia coli*, *Staphylococcus aureus*, and *Clostridium perfringens*.

The extracts with the highest concentration of total phenolic compounds were obtained using 60% DES under optimized conditions (130 °C, 20 min using microwave-assisted extraction). These extracts were subsequently subjected to antimicrobial analysis via the disk diffusion method (Figure 3).

The antimicrobial analysis ANOVA tests of the extracts revealed significant differences in the activity inhibition of *E. coli* (F = 1828.9, 4 df, *p* < 0.001), *S. aureus* (F = 887.7, 4 df, *p* < 0.001), and *C. perfringens* (F = 985.6, 4 df, *p* < 0.001), depending on the type of solvent and the algae species. The results are summarized in Table 5.

All tested extracts of *L. hyperborea* and *A. nodosum* demonstrated notable antimicrobial activity against *E. coli*, *S. aureus*, and *C. perfringens*. Interestingly, the DESs alone, used as a control, also inhibited microbial growth, which suggests a possible synergistic interaction between the DESs and the algal extracts that may enhance their antimicrobial potency. This phenomenon has been observed in previous studies, where DES components such as choline chloride have demonstrated inherent antimicrobial properties [59].

The antimicrobial effectiveness of these extracts correlates well with their phenolic content, as phenolic compounds are known for their bactericidal and bacteriostatic properties [60]. Additionally, studies have reported that certain DES formulations can promote the extraction of bioactive compounds and simultaneously exhibit antimicrobial activity [49]. Nevertheless, to disentangle the contributions of DESs and bioactive compounds to the observed activity, further research involving purified fractions, as discussed earlier, is necessary.

Following the antimicrobial assays, it was evident that the crude extracts demonstrated significant activity; however, further refinement was necessary to minimize potential interference from DES components. To address this, the extracts underwent purification using ethyl acetate, a solvent with a high affinity for polyphenols, which effectively isolated hydrophilic DES components into the aqueous layer that was subsequently discarded. This step allowed the recovery of bioactive compounds in a cleaner, more concentrated fraction [61,62]. Ethyl acetate was selected due to its proven efficiency in extracting bioactive compounds such as phenolic acids and flavonoids from plant and algal matrices, enhancing the purity of the extracts for further analysis and applications [62]. The resulting fraction was concentrated to 250 µg/mL, ensuring that bioactive compounds were the primary contributors to the observed biological activities, rather than residual DES components.

The antimicrobial activity of the extracts from *L. hyperborea* and *A. nodosum* was assessed against *C. perfringens*, *S. aureus*, and *E. coli*. Both algae species demonstrated significant antimicrobial activity, with *L. hyperborea* exhibiting the strongest inhibition, particularly against *C. perfringens* (Table 5).

For both *S. aureus* and *E. coli*, the inhibition zone diameters were relatively similar across the different extraction conditions. *A. nodosum* showed a slightly stronger inhibition against *S. aureus* (18.55 mm for AcNa/LA) compared to *L. hyperborea* (16.10 for ChCl/LA). In addition, *A. nodosum* has a more potent antimicrobial effect against *E. coli*, with zone diameters of 15.01 mm for ChCl/LA, while no more than 13.44 mm is observed with AcNa/LA and *L. hyperborean.*

**Figure 4:**
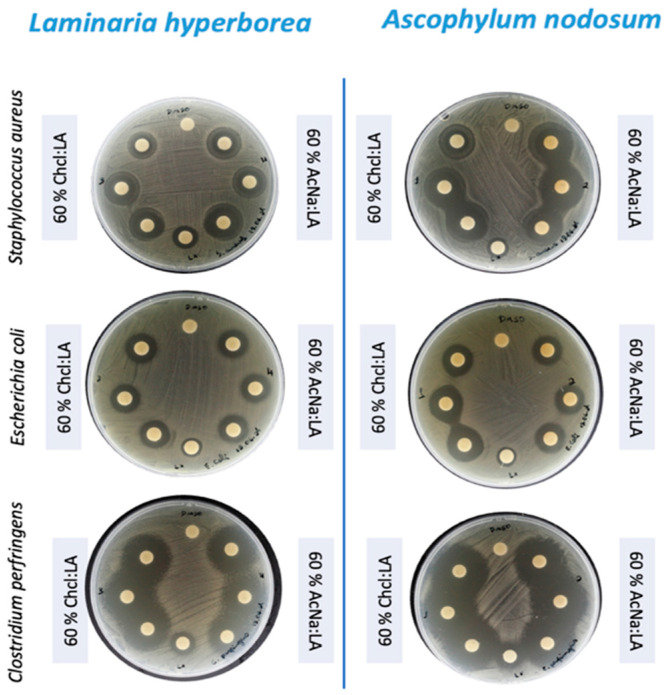
Inhibition zones of *Laminaria hyperborea* and *Ascophyllum nodosum* extracts against *S. aureus*, *E. coli*, and *C. perfringens* in disk diffusion assays.

The most striking result was the inhibition of *C. perfringens*, where both algae species exhibited significant inhibition, with zone diameters exceeding 20 mm (WT ≥ 20) for all tested conditions (Figure 4). This result is noteworthy, as *C. perfringens* is a major cause of foodborne illness and has been linked to more severe infections such as gas gangrene. The effectiveness of these algal extracts against *C. perfringens* highlights their potential as antimicrobial agents in food safety applications.

The antimicrobial effects observed in this study can be attributed to the high concentration of phenolic compounds present in the algal extracts. Phenolic compounds, including flavonoids, tannins, and hydroxybenzoic acids, have been widely recognized for their broad-spectrum antimicrobial properties [60]. These compounds typically exert their antimicrobial effects through mechanisms such as the disruption of bacterial cell membranes, the inhibition of essential enzymes, and the chelation of metal ions required for bacterial growth [63,64].

The stronger inhibitory effect observed with *A. nodosum* extracts is consistent with previous studies that reported a higher phenolic content in this species compared to *L. hyperborea* [65] and found that *A. nodosum* exhibited higher antioxidant activity, which they attributed to its elevated phenolic content. These findings suggest that the higher antimicrobial activity of *A. nodosum* could be linked to its more abundant phenolic compounds, particularly those known for antimicrobial properties, such as flavonoids and phenolic acids.

The observed antimicrobial efficacy is particularly significant in light of the growing global issue of antibiotic resistance. *S. aureus* and *E. coli* are well-known for their ability to develop resistance to multiple antibiotics, including methicillin-resistant *S. aureus* (MRSA) and multidrug-resistant *E. coli* [66]. The effectiveness of these algal extracts against these pathogens suggests that they may serve as potential natural alternatives or adjuncts to conventional antibiotics, especially in combating resistant bacterial strains [67,68].

The inhibitory activity observed against *C. perfringens* is particularly relevant in food safety, as this pathogen is responsible for numerous foodborne illnesses [69]. The ability of *L. hyperborea* and *A. nodosum* extracts to inhibit *C. perfringens* highlights their potential as natural preservatives, offering a sustainable and eco-friendly alternative to synthetic antimicrobial agents commonly used in the food industry.

Phenolic compounds, a diverse group of secondary metabolites found in brown algae, have demonstrated broad-spectrum antimicrobial activity. Their mechanisms of action are varied and complex, including the disruption of cell membranes and the inhibition of enzyme activity essential for bacterial growth [3]. The stronger inhibitory effect observed with *A. nodosum* extracts aligns with previous studies reporting a higher phenolic content in this species compared to *L. hyperborea*. For instance, Blanco-Pascual et al. [65] discuss the antioxidant properties of films developed from unrefined extracts of these two species, noting the higher antioxidant activity in *A. nodosum* films, which could be related to phenolic content. While this study focuses on antioxidant activity, it highlights the potential differences in bioactive compound concentrations between the two species.

The observed antimicrobial activity against *C. perfringens*, *S. aureus*, and *E. coli* is particularly relevant given the increasing prevalence of antibiotic resistance in these pathogens. *C. perfringens* is a major cause of foodborne illness and can also cause gas gangrene, while *S. aureus* and *E. coli* are common causes of various infections, including skin infections, pneumonia, and urinary tract infections. The effectiveness of the algal extracts against these bacteria suggests their potential as natural antimicrobial agents in various applications, including food preservation and pharmaceutical development.

Further investigation is warranted to identify the specific phenolic compounds responsible for the observed antimicrobial activity and to elucidate their mechanisms of action. Additionally, studies evaluating the efficacy of these extracts against other clinically relevant pathogens, including drug-resistant strains, would be valuable. Investigating the potential synergistic effects of combining these extracts with existing antibiotics could also lead to the development of novel therapeutic strategies. The removal of DES improved the reliability and reproducibility of bioactivity assessments, particularly in antimicrobial tests by reducing background noise and potential antagonistic effects, thereby enhancing the extracts’ effectiveness.

### 3.6. Characterization of Residues Resulting from the Extraction of Bioactive Compounds

The characterization of the solid residues from *A. nodosum* and *L. hyperborea*, obtained after the extraction of bioactive compounds using deep eutectic solvents (DESs) assisted by microwaves, revealed distinct behaviors in their chemical, thermal, and structural properties.

The chemical composition of the seaweed residues obtained after treatment with DESs and microwave assistance reveals distinct differences between the two species, *A. nodosum* and *L. hyperborea*, particularly in their glucan and acid-insoluble residue content. The ANOVA tests revealed significant differences in the extraction efficiency of the polysaccharides, namely glucan (F = 637.7, 8 df, *p* < 0.001), xylan/galactan/mannan (F = 312.5, 8 df, *p* < 0.001), fucoidan (F = 46.7, 8 df, *p* < 0.001), and the acid-insoluble residue (F = 21,380.7, 8 df, *p* < 0.001), depending on the type of solvent, the time/temperature combination, and the algae species. The results are summarized in Table A8 (factors), Table A9 (first-order interactions), and Table 6 (second-order interactions).

The glucan content in the residue from *A. nodosum* (9.64 g/100 g) was significantly lower than that observed for *L. hyperborea* (17.47 g/100 g). This difference can be attributed to the higher concentration of glucans, particularly cellulose and other glucose-based polymers, present in the *L. hyperborea* species. *L. hyperborea*, known for its higher content of polysaccharides, like alginates, also contains a considerable amount of glucan, which is a critical energy source and contributes to the structural integrity of the algae [70]. In contrast, *A. nodosum*, although rich in alginates, contains a lower amount of glucan, reflecting the species’ different biochemical composition, where polysaccharides such as fucoidan are more predominant. These findings align with previous research suggesting that glucan levels can vary significantly across different algal species, depending on their biochemical makeup [71].

The levels of xylan/galactan/mannan were also lower in *A. nodosum*, which contained 2.75 g/100 g, and *L. hyperborea*, which showed 3.71 g/100 g. These polysaccharides, which are part of the hemicellulose fraction, exhibit some variation but are not drastically different between the two species. This indicates that both *A. nodosum* and *L. hyperborea*. have comparable amounts of hemicellulose, a polysaccharide component that plays an essential role in the structural properties of algae, contributing to the overall mechanical strength and flexibility [72]. The slight difference in content could be linked to species-specific variations in the hemicellulosic matrix or the extraction process using DESs, which might have influenced the solubility and extraction efficiency of these components.

The characterization of the solid residues obtained after the microwave-assisted extraction using deep eutectic solvents (DESs) revealed comparable levels of fucoidan in *A. nodosum* (1.43 g/100 g) and *L. hyperborea* (1.35 g/100 g). Fucoidan, a sulfated polysaccharide primarily composed of L-fucose and sulfate esters, is a major bioactive constituent of brown seaweeds, and its presence in the post-extraction residues highlights the partial retention of functional biopolymers even after solvent treatment [73].

Although both species showed relatively low but similar residual fucoidan contents, previous studies have demonstrated that *A. nodosum* generally contains higher initial fucoidan concentrations than *L. hyperborea*, which may reflect more efficient extraction or structural differences influencing solubilization [70,74]. The structural complexity and branching of fucoidan chains, along with interactions with other matrix components like alginate and cellulose, can impact extraction yields and residue composition [74].

The acid-insoluble residue content is significantly higher in *A. nodosum* (54.61 g/100 g) compared to *L. hyperborea* (18.78 g/100 g). This difference indicates that *A. nodosum* has a higher proportion of lignocellulosic materials that are resistant to acidic hydrolysis, which is likely composed of more recalcitrant biomass such as lignin or other complex polymers. The higher acid-insoluble residue content in *A. nodosum* suggests that the species has a more lignified structure, which could explain the increased resistance to chemical breakdown during the DES treatment. On the other hand, *L. hyperborea*, being a brown alga with a higher content of alginates, exhibits lower amounts of acid-insoluble residues, as alginates are generally more amenable to degradation and solubilization during the extraction process [75].

The differences in the chemical composition between *A. nodosum* and *L. hyperborea* residues after DES treatment suggest potential variations in their applications. The higher glucan content in *L. hyperborea* suggests it may be more suitable for bioenergy production, particularly for fermentation processes that utilize glucan-based carbohydrates. The lower acid-insoluble residue content in *L. hyperborea* also implies that it may be easier to process into value-added products such as biofuels or biochemicals. In contrast, the higher acid-insoluble residue content of *A. nodosum* indicates that it could be more suitable for applications where structural integrity is desired, such as in the production of bioplastics or other lignocellulosic-based materials [76].

Both algae show promise for a variety of applications, depending on their biochemical composition, which highlights the importance of selecting the right species for specific industrial purposes.

### 3.7. Thermal Analysis (DSC and TGA), X-Ray Diffraction (XRD), and Scanning Electron Microscopy (SEM)

**Figure 5:**
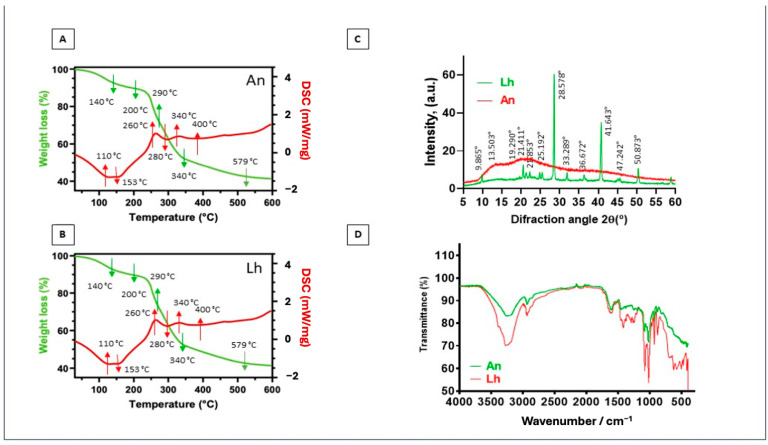
Thermal analysis (differential scanning calorimetry (DSC) and thermogravimetric analysis (TGA)) and structural X-ray diffraction. Analyses of *A. nodosum* (An) and *L. hyperborea* (Lh) residues after extraction with deep eutectic solvents (DESs). (**A**) DSC and TGA thermogram of *A. nodosum* (An) residues; (**B**) DSC thermogram of *L. hyperborea* (Lh) residues; (**C**) X-ray diffraction (XRD) pattern of *A. nodosum* (An) and *L. hyperborea* (Lh) residues; (**D**) FT-IR analysis pattern of *A. nodosum* (An) and *L. hyperborea* (Lh) residues.

Figure 5A,B present the characteristic thermal profiles of the solid residues. The residues underwent four distinct stages of weight loss during heating, each associated with different decomposition processes of their lignocellulosic components.

In the first stage (up to 140 °C), a mass loss of approximately 10% was observed [77], attributed to the evaporation of water and physically adsorbed moisture. This behavior was confirmed by the DSC curve, which revealed an initial endothermic reaction at around 100 °C, corresponding to the removal of residual moisture from the sample. The second stage (200–290 °C) showed a significant mass loss (~20%), mainly due to the decomposition of hemicelluloses and pectins, which are more thermally degradable [78,79]. In the third stage (300–380 °C), cellulose degradation predominated, evidenced by an endothermic reaction recorded at 280 °C. Additionally, exothermic reactions were observed at 260 °C, 340 °C, and 400 °C, indicating the oxidation of more resistant lignocellulosic components [80]. Finally, in the fourth stage (above 380 °C), combustion of fixed carbon occurred, concluding the thermal process at approximately 579 °C. In this stage, the accumulated mass loss was approximately 45%. These results highlight the complexity of the lignocellulosic composition in the residues and underscore their potential as a source of bioenergy. The X-ray diffraction (XRD) patterns (Figure 5C,D) provided complementary information about the structure of the solid residues. The results revealed significant differences between the residues of *A. nodosum* and *L. hyperborea*.

The *A. nodosum* residues exhibited predominantly amorphous characteristics, suggesting the breakdown of crystalline components during the extraction process. In contrast, the *L. hyperborea* residues displayed a predominantly crystalline structure, with several well-defined diffraction peaks. These peaks were observed at 9°, 21°, 25°, 33°, 36°, 41°, 47°, and 50° and were attributed to the presence of polysaccharides, such as alginates, which are abundant in brown algae [81]. Furthermore, a prominent peak at 28° was identified in the *L. hyperborea* residues, corresponding to the presence of calcium, often associated with alginate–calcium complexes [81]. The crystalline structure observed in *L. hyperborea* reflects its higher content of mineral and polysaccharide components, while the amorphous structure of *A. nodosum* suggests greater degradation of these compounds during the treatment. The residues of *A. nodosum* and *L. hyperborea* displayed distinct thermal and structural behaviors, reflecting differences in their chemical compositions. The thermal analysis revealed that both residues have significant energy potential due to the presence of fixed carbon, while the structural analysis highlighted the preservation of mineral and polysaccharide compounds in *L. hyperborea*. These findings suggest that *L. hyperborea* residues have potential applications in areas such as adsorbents, biomaterials, and bioenergy, due to their crystalline structure and abundance of alginates. On the other hand, the amorphous *A. nodosum* residues may be more suitable for applications requiring greater chemical reactivity or easier processing.

**Figure 6:**
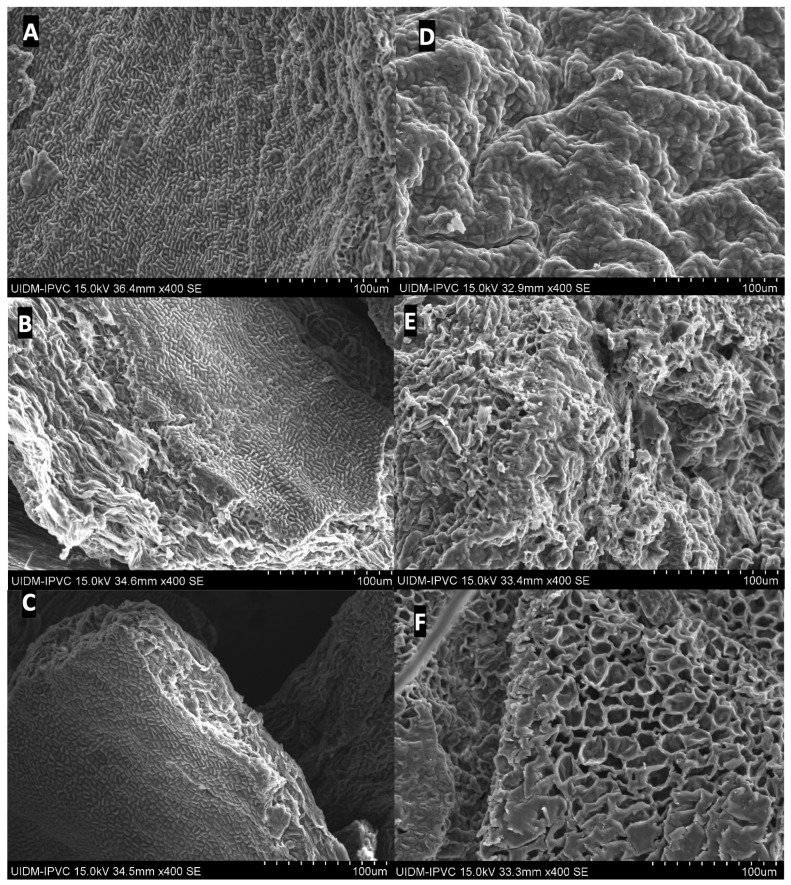
Scanning electron microscopy (SEM) images of *Laminaria hyperborea* (Lh) and *Ascophyllum nodosum* (An) before and after treatment with deep eutectic solvents (DESs) assisted by microwave heating. (**A**) Lh in nature; (**B**) Lh treated with DES at 130 °C for 20 min; (**C**) Lh treated with DES at 150 °C for 10 min; (**D**) An in nature; (**E**) An treated with DES at 130 °C for 20 min; (**F**) An treated with DES at 150 °C for 10 min.

Scanning electron microscopy (SEM) was performed to assess the morphological changes in the macroalgae *Laminaria hyperborea* (Lh) and *Ascophyllum nodosum* (An) residues following treatment with deep eutectic solvents (DESs) assisted by microwave heating. This analysis aimed to elucidate the physical disruptions induced by the extraction process and their relation to the release efficiency of bioactive compounds. The SEM micrographs (Figure 6) reveal substantial differences between untreated samples and post-extraction residues. Figure 6A (Lh) and 6D (An) correspond to the in-nature, untreated biomass, displaying relatively smooth, continuous surfaces with an intact external coating—typical of unaltered algal cell walls. In contrast, samples treated at 130 °C for 20 min (Figure 6B,E) show noticeable surface degradation. The appearance of rough textures, fissures, and disorganization indicates a partial breakdown of the outer layers. Localized structural collapse and irregular formations suggest that the DES and thermal treatment initiated cell wall disruption, facilitating compound release.

These effects are more pronounced in the samples treated at 150 °C for 10 min (Figure 6C,F), which exhibit significant structural collapse. Large cavities, fragmented surfaces, and almost complete loss of the superficial protective layer are evident. Such severe morphological damage indicates effective matrix disruption caused by the combined action of heat and DESs, supporting the high extraction yields observed.

Additionally, the presence of lighter-toned areas on the surface may correspond to exposed inner fibers or the removal of external impurities, further confirming the structural impact of the treatment. These morphological modifications are directly associated with enhanced permeability and improved extraction performance, reinforcing the effectiveness of the proposed green extraction strategy.

The results obtained in this study demonstrate the considerable potential of *Laminaria hyperborea* and *Ascophyllum nodosum* as sustainable sources of bioactive compounds, particularly phenolic compounds with notable antioxidant and antimicrobial activities. FT-IR analysis confirmed the chemical complexity of the extracts, highlighting functional groups such as phenols, carbohydrates, and lipids, which are crucial for their biological effects. The antimicrobial efficacy of the crude extracts, especially against foodborne pathogens like *Clostridium perfringens*, was strongly correlated with the phenolic content extracted under optimized microwave-assisted DES conditions. Moreover, the synergistic interaction between the DESs and algal compounds not only improved extraction efficiency but also enhanced antimicrobial effects. Purification using ethyl acetate further concentrated phenolics, allowing more precise bioactivity evaluation. The contrasting chemical profiles and structural features of the residual biomass from both species suggest diverse valorization routes, including bioenergy production for *L. hyperborea* due to its higher glucan content and bioplastic applications for *A. nodosum* given its lignocellulosic-rich, acid-insoluble residues. This integrated approach aligns with green chemistry principles and supports the biorefinery concept, promoting the full utilization of marine biomass with minimal environmental impact. Together, these findings lay a solid foundation for future investigations into the specific bioactive compounds responsible for antimicrobial action, their synergistic mechanisms, and the optimization of extraction and purification processes to harness the full industrial and pharmaceutical potential of these macroalgae.

These characterization results offer valuable insights for downstream applications of the residual biomass. The FT-IR analysis confirmed the presence of functional groups such as phenols, carbohydrates, and lipids, which are linked to antioxidant, antimicrobial, and potential therapeutic activities. The structural integrity observed in *L. hyperborea* residues through XRD, particularly the retention of crystalline regions associated with alginate–calcium complexes, suggests their suitability for materials science applications such as bio-based adsorbents or biodegradable composites. In contrast, the more amorphous structure of *A. nodosum* residues may be advantageous for processes requiring higher reactivity, such as the production of bioplastics. SEM images revealed that microwave-assisted DES extraction effectively disrupted the cell wall structure, especially at higher temperatures, enhancing extraction yields and facilitating further processing. Thermal analysis (DSC and DTG) confirmed that both algal residues possess significant fixed carbon content and exhibit multi-stage decomposition, underscoring their potential as feedstocks for bioenergy production. Together, these findings support the integration of these algae into a sustainable biorefinery model, where both the bioactive extracts and the structurally characterized residues are efficiently utilized for high-value applications.

## 4. Conclusions

This study highlights the potential of *Laminaria hyperborea* and *Ascophyllum nodosum* as valuable and sustainable sources of bioactive compounds, particularly phenolics with demonstrated antioxidant and antimicrobial activities. The use of deep eutectic solvents (DESs) in combination with microwave-assisted extraction (MAE) proved to be an effective and environmentally friendly strategy for obtaining high-value extracts from brown macroalgae. Among the tested conditions, the extract obtained from *L. hyperborea* exhibited the strongest antimicrobial effect, particularly against *Clostridium perfringens*, underscoring its potential for applications in food safety or pharmaceutical preservation. Fourier transform infrared (FT-IR) spectroscopy confirmed the presence of structurally diverse compounds, including phenolics, sulfated polysaccharides, and proteins. Furthermore, the use of ethyl acetate purification significantly enhanced the clarity and reproducibility of the bioactivity assays, suggesting its importance in standardizing extract evaluation protocols. Overall, the results support the development of green and sustainable extraction processes for the valorization of marine biomass within the framework of the circular bioeconomy. These findings open promising perspectives for the integration of macroalgal-derived extracts in functional food, nutraceutical, or cosmeceutical applications, promoting both resource efficiency and environmental sustainability.

## Figures and Tables

**Table 1:** Chemical composition and mineral content of the brown algae studied (g of component per 100 g of raw material, oven-dry basis).

Components	*p*-Value	*Laminaria hyperborea*	*Ascophyllum nodosum*
Ash	NS	33.80 ± 0.07	24.25 ± 0.18
Crude protein	0.05	11.55 ± 0.03	8.73 ± 0.05
% water extraction	0.05	56.18 ± 0.77	51.98 ± 0.05
Rhamnose	0.05	9.35 ± 0.17	3.05 ± 0.10
% ethanol extraction	NS	10.96 ± 1.00	14.93 ± 1.56
Glucan	0.05	6.11 ± 0.42	2.48 ± 0.11
Xylan/galactan/mannan	NS	1.43 ± 0.14	1.41 ± 0.45
Fucoidan	NS	1.46 ± 0.19	3.20 ± 0.57
Acid-insoluble residue	0.05	5.00 ± 0.51	12.63 ± 0.25

Notes: Statistical significance was set at *p*  <  0.05; NS—non-significant.

**Table 2:** Mean results ± standard error (second-order interactions) of the preliminary extraction contents for total phenolic content (TPC) and antioxidant activity: 2,2-Diphenyl-1-picrylhydrazyl (DPPH) radical scavenging activity and 2,2′-azino-bis (3-ethylbenzothiazoline-6-sulfonic acid) (ABTS) radical cation decolorization assay. The significant differences were tested with a full factorial ANOVA. The homogeneity of variances was confirmed via Levene’s test (*p* > 0.05). The normal distribution of residuals was confirmed with a Shapiro–Wilk test (*p* > 0.05).

Algae × S(%) × Solvent	TPC	DPPH	ABTS
	*p* < 0.001	*p* < 0.001	*p* > 0.05
A.n. × 100 × ChCl/AL	5.41 ± 0.46 ^e^	13.09 ± 0.03 ^b^	0.22 ± 0.02
L.h. × 100 × ChCl/AL	2.61 ± 0.46 ^a^	12.95 ± 0.03 ^a^	0.16 ± 0.02
A.n. × 80 × ChCl/AL	7.32 ± 0.46 ^f^	13.26 ± 0.03 ^f^	0.22 ± 0.02
L.h. × 80 × ChCl/AL	3.22 ± 0.46 ^c^	13.14 ± 0.03 ^c^	0.17 ± 0.02
A.n. × 60 × ChCl/AL	10.28 ± 0.46 ^g^	13.18 ± 0.03 ^d^	0.20 ± 0.02
L.h. × 60 × ChCl/AL	2.65 ± 0.46 ^b^	13.48 ± 0.03 ^g^	0.17 ± 0.02
A.n. × 100 × AcNa/AL	5.69 ± 0.46 ^d^	13.04 ± 0.03 ^b^	0.26 ± 0.02
L.h. × 100 × AcNa/AL	6.18 ± 0.46 ^e^	13.17 ± 0.03 ^e^	0.35 ± 0.02
A.n. × 80 × AcNa/AL	4.13 ± 0.46 ^d^	12.99 ± 0.03 ^b^	0.20 ± 0.02
L.h. × 80 × AcNa/AL	5.32 ± 0.46 ^d^	13.11 ± 0.03 ^c^	0.30 ± 0.02
A.n. × 60 × AcNa/AL	4.84 ± 0.46 ^d^	13.08 ± 0.03 ^b^	0.20 ± 0.02
L.h. × 60 × AcNa/AL	5.90 ± 0.46 ^d^	13.24 ± 0.03 ^e^	0.32 ± 0.02

Notes: A.n.—*A. nodosum*; L.h.—*L. hyperborea*. Solvents—choline chloride/lactic acid (ChCl/LA) and sodium acetate/lactic acid (AcNa/LA). Units of measurement: TPC—milligrams of gallic acid equivalents per gram of dry weight (mg GAE/g DW); DPPH and ABTS—Trolox equivalents (TE) in milligrams per gram of dry weight (mg TE/g DW). Different letters in columns in superscript are indicative of significant differences after the LSD post hoc test (*p* < 0.05).

**Table 3:** Mean results ± standard error (third-order interactions) of the optimized extraction contents for total phenolic content (TPC), antioxidant activity (2,2-Diphenyl-1-picrylhydrazyl (DPPH) radical scavenging activity, 2,2′-azino-bis (3-ethylbenzothiazoline-6-sulfonic acid) (ABTS) radical cation decolorization assay, ferric reducing antioxidant power (FRAP) assay), and protein. The significant differences were tested with full factorial ANOVAs. The homogeneity of variances was confirmed with Levene’s test (*p* > 0.05). The normal distribution of the residuals was confirmed with the Shapiro–Wilk test (*p* > 0.05).

	TPC	DPPH	ABTS	FRAP	Protein
Algae × Solvent × Time × Temperature	*p* < 0.01	*p* < 0.001	*p* < 0.01	*p* > 0.05	*p* > 0.05
A.n. × ChCl/AL × 10 × 130	31.70 ± 1.34 ^e^	44.90 ± 1.48 ^e^	74.73 ± 3.55 ^h^	29.83 ± 2.36	
A.n. × ChCl/AL × 10 × 150	47.51 ± 1.34 ^g^	43.23 ± 1.48 ^e^	90.55 ± 3.55 ^j^	85.04 ± 2.36	-
A.n. × ChCl/AL × 20 × 130	50.62 ± 1.34 ^h^	48.37 ± 1.48 ^f^	83.64 ± 3.55 ^i^	82.78 ± 2.36	-
A.n. × ChCl/AL × 20 × 150	45.89 ± 1.34 ^f^	42.32 ± 1.48 ^e^	83.23 ± 3.55 ^i^	40.63 ± 2.36	-
A.n. × AcNa/AL × 10 × 130	33.29 ± 1.34 ^e^	46.66 ± 1.48 ^e^	95.10 ± 3.55 ^l^	35.41 ± 2.36	-
A.n. × AcNa/AL × 10 × 150	32.58 ± 1.34 ^e^	44.64 ± 1.48 ^e^	90.39 ± 3.55 ^j^	85.25 ± 2.36	-
A.n. × AcNa/AL × 20 × 130	33.41 ± 1.34 ^e^	47.50 ± 1.48 ^f^	93.27± 3.55 ^k^	150.45 ± 2.36	-
A.n. × AcNa/AL × 20 × 150	27.58 ± 1.34 ^d^	53.49 ± 1.48 ^g^	83.42 ± 3.55 ^i^	35.41 ± 2.36	-
L.h. × ChCl/AL × 10 × 130	15.08 ± 1.34 ^a^	31.79 ± 1.48 ^c^	12.80 ± 3.55 ^a^	41.35 ± 2.36	-
L.h. × ChCl/AL × 10 × 150	19.12 ± 1.34 ^c^	33.39 ± 1.48 ^c^	18.54 ± 3.55 ^b^	52.86 ± 2.36	-
L.h. × ChCl/AL × 20 × 130	15.34 ± 1.47 ^a^	35.13 ± 1.62 ^c^	28.44 ± 3.88 ^c^	53.20 ± 2.58	-
L.h. × ChCl/AL × 20 × 150	17.64 ± 1.24 ^b^	35.59 ± 1.37 ^c^	52.68 ± 3.28 ^g^	48.82 ± 2.18	-
L.h. × AcNa/AL × 10 × 130	12.56 ± 1.34 ^a^	27.16 ± 1.48 ^b^	34.02 ± 3.55 ^d^	48.44 ± 2.36	-
L.h. × AcNa/AL × 10 × 150	13.09 ± 1.34 ^a^	38.27 ± 1.48 ^d^	41.51 ± 3.55 ^f^	105.72 ± 2.36	-
L.h. × AcNa/AL × 20 × 130	13.62 ± 1.34 ^a^	28.19 ± 1.48 ^b^	38.85 ± 3.55 ^e^	69.64 ± 2.36	-
L.h. × AcNa/AL × 20 × 150	13.09 ± 1.34 ^a^	25.80 ± 1.48 ^a^	29.00 ± 3.55 ^c^	48.61 ± 2.36	-

Notes: A.n.—*A. nodosum*; L.h.—*L. hyperborea*. Solvents—choline chloride/lactic acid (ChCl/LA) and sodium acetate/lactic acid (AcNa/LA). Units of measurement: TPC—milligrams of gallic acid equivalents per gram of dry weight (mg GAE/g DW); DPPH and ABTS—Trolox equivalents (TE) in milligrams per gram of dry weight (mg TE/g DW); FRAP—g of ferrous equivalents (FE) per 100 g of dry weight (g FE/100 g DW); protein—g of bovine serum albumin equivalents (BSA) per of dry weight (g BSA/g DW). Different letters in columns in superscript are indicative of significant differences after the LSD post hoc test (*p* < 0.05).

**Table 4:** Mean results ± standard error (second-order interaction) of the optimized extraction contents of the different phenolic compounds: 3,4-dihydroxybenzoic acid (DHB), phthalic acid (PHT), salicylic acid (SAL), 4-hydroxybenzoic acid (HBA), and vanillin (VAN). The significant differences were tested with full factorial ANOVAs. The homogeneity of variances was confirmed with Levene’s test (*p* > 0.05). The normal distribution of the residuals was confirmed with the Shapiro–Wilk test (*p* > 0.05).

	DHB	PHT	SAL	HBA	VAN
Algae × Solvent × TT	*p* < 0.001	*p* < 0.001	*p* < 0.01	*p* > 0.05	*p* < 0.001
A.n. × ChCl/LA × 20,130	6.58 ± 7.4 ^a,b^	33.99 ± 5.98 ^b^	2.57 ± 0.30 ^c^	7.55 ± 0.20	84.25 ± 58.54 ^a^
A.n. × ChCl/LA × 10,150	50.71 ± 7.4 ^d^	33.42 ± 5.98 ^b^	1.98 ± 0.30 ^c^	6.23 ± 0.20	180.25 ± 58.54 ^a^
A.n. × AcNa/LA × 20,130	30.25 ± 7.4 ^c^	56.51 ± 5.98 ^c^	2.46 ± 0.30 ^c^	8.83 ± 0.20	613.50 ± 58.54 ^b^
A.n. × AcNa/LA × 10,150	678.05 ± 7.4 ^f^	31.29 ± 5.98 ^b^	3.78 ± 0.30 ^d^	8.63 ± 0.20	3981.00 ± 58.54 ^d^
L.h. × ChCl/LAx 20,130	1.12 ± 7.4 ^a^	126.43 ± 5.98 ^d^	≈0 ^a^	7.43 ± 0.20	66.56 ± 58.54 ^a^
L.h. × ChCl/LA × 10,150	9.80 ± 7.4 ^b^	38.42 ± 5.98 ^b^	0.61± 0.30 ^b^	6.09 ± 0.20	81.82 ± 58.54 ^a^
L.h. × AcNa/LA × 20,130	30.17 ± 7.4 ^c^	26.71 ± 5.98 ^a^	2.45 ± 0.30 ^c^	7.94 ± 0.20	1111.50 ± 58.54 ^c^
L.h. × AcNa/LA × 10,150	505.10 ± 7.4 ^e^	46.72 ± 5.98 ^b^	2.35 ± 0.30 ^c^	8.35 ± 0.20	6718.50 ± 58.54 ^e^

Notes: TT—time/temperature combination (min, °C). A.n.—*A. nodosum*; L.h.—*L. hyperborea.* Solvents—choline chloride/lactic acid (ChCl/LA) and sodium acetate/lactic acid (AcNa/LA). Unit of measurement: µg/g dry weight (µg/g DW). Different letters in superscript in the same column are indicative of significant differences after the LSD post hoc test (*p* < 0.05).

**Table 5:** Mean results ± standard error of the measurements of the inhibition zones for the different microorganisms: *Escherichia coli* (*E. coli*), *Staphylococcus aureus* (*S. aureus*), and *Clostridium perfringens* (*C. perfringens*). The significant differences were tested with full factorial ANOVAs. The homogeneity of variances was confirmed with Levene’s test (*p* > 0.05). The normal distribution of the residuals was confirmed with the Shapiro–Wilk test (*p* > 0.05).

	*E. coli*	*S. aureus*	*C. perfringens*
Solvent	*p* < 0.05	*p* > 0.05	*p* > 0.05
ChCl/LA	14.26 ± 0.23	16.70 ± 0.40	24.35 ± 0.54
AcNa/LA	13.52 ± 0.23	17.25 ± 0.40	23.62 ± 0.54
Algae	*p* < 0.05	*p* > 0.05	*p* < 0.05
*L. hyperborea*	13.47 ± 0.23	16.63 ± 0.40	22.68 ± 0.54
*A. nodosum*	14.31 ± 0.23	17.32 ± 0.40	25.30 ± 0.54
Solvent × algae	*p* < 0.05	*p* < 0.01	*p* > 0.05
ChCl/LA × *A. nodosum*	15.01 ± 0.33 ^b^	16.10 ± 0.57 ^a^	25.34 ± 0.77 ^b^
AcNa/LA × *A. nodosum*	13.60 ± 0.33 ^a^	18.55 ± 0.57 ^b^	25.25 ± 0.77 ^b^
ChCl/LA × *L. hyperborea*	13.50 ± 0.33 ^a^	17.31 ± 0.57 ^a^	23.37 ± 0.77 ^b^
AcNa/LA × *L. hyperborea hyperborea*	13.44 ± 0.33 ^a^	15.95 ± 0.57 ^a^	21.99 ± 0.77 ^a^

Notes: Solvents—choline chloride/lactic acid (ChCl/LA) and sodium acetate/lactic acid (AcNa/LA). Unit of measurement: mm. Different letters in superscript in the same column are indicative of significant differences after the LSD post hoc test (*p* < 0.05).

**Table 6:** Mean results (second-order interaction) of the optimised extraction residue polysaccharides glucan (GLU), xylan/galactan/mannan (XGM), fucoidan (FUC), and acid insoluble residue (AIR). The significant differences were tested with full factorial ANOVAs. The homogeneity of variances was confirmed with Levene’s test (*p* > 0.05). The normal distribution of the residuals was confirmed with the Shapiro-Wilks test (*p* > 0.05).

	GLU	XGM	FUC	AIR
Algae × Solvent × TT	*p* > 0.05	*p* < 0.001	*p* > 0.05	*p* > 0.05
A.n. × ChCl:AL × 20,130	8.57	3.06 ^b^	1.86	29.97
A.n. × ChCl:AL × 10,150	11.75	3.52 ^b^	2.46	64.23
A.n. × AcNa:AL × 20,130	9.80	3.10 ^b^	1.40	45.29
A.n. × AcNa:AL × 10,150	8.45	1.33 ^a^	≈0	78.96
L. h. × ChCl:AL × 20,130	19.73	3.20 ^b^	2.24	22.06
L. h. × ChCl:AL × 10,150	20.30	3.23 ^b^	1.58	22.52
L. h. × AcNa:AL × 20,130	15.47	2.83 ^b^	1.58	14.61
L. h. × AcNa:AL × 10,150	14.38	5.59 ^c^	≈0	15.94

Notes: TT—Combination Time Temperature (min, °C). Solvents—choline chloride:lactic acid (ChCl:LA) and sodium acetate:lactic acid (AcNa:LA). Unit of measurement g/100g or percentage. Different letters in superscript in the same column are indicative of significant differences after the LSD post hoc test (*p* < 0.05).

## Data Availability

The original contributions presented in the study are included in the article, further inquiries can be directed to the corresponding authors.

## Appendix A

### Appendix A.1. Complementary Tables for Section 3.2 Preliminary Extraction

**Table A1:** Mean results ± standard error (factors) of the preliminary extraction contents for total phenolic content (TPC) and antioxidant activity: 2,2-Diphenyl-1-picrylhydrazyl (DPPH) radical scavenging activity and 2,2′-azino-bis (3-ethylbenzothiazoline-6-sulfonic acid) (ABTS) radical cation decolorization assay. The significant differences were tested with a full factorial ANOVA. The homogeneity of variances was confirmed with Levene’s test (*p* > 0.05). The normal distribution of the residuals was confirmed with the Shapiro–Wilk test (*p* > 0.05).

	TPC	DPPH	ABTS
Solvent (%)	*p* < 0.01	*p* < 0.001	*p* < 0.05
60	5.92 ± 0.23 ^b^	13.25 ± 0.02 ^c^	0.22 ± 0.01 ^a^
80	5.0 ± 0.23 ^a^	13.12 ± 0.02 ^b^	0.22 ± 0.01 ^a^
100	4.97 ± 0.23 ^a^	13.06 ± 0.02 ^a^	0.25 ± 0.01 ^b^
Algae	*p* < 0.001	*p* < 0.001	*p* < 0.01
*L. hyperborea*	6.28 ± 0.19	13.18 ± 0.01	0.25 ± 0.01
*A. nodosum*	4.31 ± 0.19	13.11 ± 0.01	0.22 ± 0.01
Solvent	*p* > 0.05	*p* < 0.001	*p* < 0.001
AcNa/AL	5.34 ± 0.19	13.11 ± 0.01	0.27 ± 0.01
ChCl/AL	5.25 ± 0.19	13.18 ± 0.01	0.19 ± 0.01

Notes: Solvents—choline chloride/lactic acid (ChCl/LA) and sodium acetate/lactic acid (AcNa/LA). Units of measurement: TPC—milligrams of gallic acid equivalents per gram of dry weight (mg GAE/g DW); DPPH and ABTS—Trolox equivalents (TE) in milligrams per gram of dry weight (mg TE/g DW. Different letters in columns with more than two factor levels in superscript are indicative of significant differences after the LSD post hoc test (*p* < 0.05).

**Table A2:** Mean results ± standard error (first-order interactions) of the preliminary extraction contents for total phenolic content (TPC) and antioxidant activity: 2,2-Diphenyl-1-picrylhydrazyl (DPPH) radical scavenging activity and 2,2′-azino-bis (3-ethylbenzothiazoline-6-sulfonic acid) (ABTS) radical cation decolorization assay. The significant differences were tested with a full factorial ANOVA. The homogeneity of variances was confirmed via Levene’s test (*p* > 0.05). The normal distribution of residuals was confirmed via the Shapiro–Wilk test (*p* > 0.05).

	TPC	DPPH	ABTS
Algae × Solvent	*p* < 0.001	*p* < 0.01	*p* < 0.001
A.n. × ChCl/AL	7.67 ± 0.27 ^d^	13.18 ± 0.02 ^b^	0.21 ± 0.01 ^b^
A.n. × AcNa/AL	4.83 ± 0.27 ^b^	13.04 ± 0.02 ^a^	0.22 ± 0.01 ^b^
L.h. × ChCl/AL	2.83 ± 0.27 ^a^	13.19 ± 0.02 ^b^	0.17 ± 0.01 ^a^
L.h. × AcNa/AL	5.80 ± 0.27 ^c^	13.17 ± 0.02 ^b^	0.33 ± 0.01 ^c^
Solvent × S (%)	*p* < 0.01	*p* < 0.001	*p* < 0.05
ChCl/AL × 60	6.46 ± 0.33 ^b^	13.33 ± 0.02 ^c^	0.18 ± 0.01 ^a^
AcNa/AL × 60	5.37 ± 0.33 ^a^	13.16 ± 0.02 ^b^	0.26 ± 0.01 ^b^
ChCl/AL × 80	5.27 ± 0.33 ^b^	13.20 ± 0.02 ^b^	0.19 ± 0.01 ^a^
AcNa/AL × 80	4.70 ± 0.33 ^a^	13.05 ± 0.02 ^a^	0.25 ± 0.01 ^b^
ChCl/AL × 100	4.01 ± 0.33 ^a^	13.02 ± 0.02 ^a^	0.19 ± 0.01 ^a^
AcNa/AL × 100	5.94 ± 0.33 ^b^	13.10 ± 0.02 ^b^	0.31 ± 0.01 ^c^
Algae × S (%)	*p* < 0.001	*p* < 0.001	*p* > 0.05
A.n. × 60	7.56 ± ^e^	13.13 ± 0.02 ^b^	0.20 ± 0.01
A.n. × 80	5.72 ± 0.33 ^d^	13.12 ± 0.02 ^a^	0.21 ± 0.01
A.n. × 100	5.55 ± 0.33 ^c^	13.06 ± 0.02 ^a^	0.24 ± 0.01
L.h. × 60	4.27 ± 0.33 ^a^	13.34 ± 0.02 ^c^	0.25 ± 0.01
L.h. × 80	4.27 ± 0.33 ^a^	13.12 ± 0.02 ^b^	0.24 ± 0.01
L.h. × 100	4.39 ± 0.33 ^b^	13.06 ± 0.02 ^a^	0.26 ± 0.01

Notes: A.n.—*A. nodosum*; L.h.—*L. hyperborea*. Solvents—choline chloride/lactic acid (ChCl/LA) and sodium acetate/lactic acid (AcNa/LA). Units of measurement: TPC—milligrams of gallic acid equivalents per gram of dry weight (mg GAE/g DW); DPPH and ABTS—Trolox equivalents (TE) in milligrams per gram of dry weight (mg TE/g DW). Different letters in columns between lines in superscript are indicative of significant differences after the LSD post hoc test (*p* < 0.05).

### Appendix A.2. Complementary Tables for Section 3.4 Optimizing Extraction Conditions for Enhanced Bioactive Compound Recovery

**Table A3:** Mean results ± standard error (factors) of the optimized extraction contents for total phenolic content (TPC), antioxidant activity (2,2-Diphenyl-1-picrylhydrazyl (DPPH) radical scavenging activity, 2,2′-azino-bis (3-ethylbenzothiazoline-6-sulfonic acid) (ABTS) radical cation decolorization assay, and ferric reducing antioxidant power (FRAP) assay), and protein. The significant differences were tested with full factorial ANOVAs. The homogeneity of variances was confirmed with Levene’s test (*p* > 0.05). The normal distribution of the residuals was confirmed with the Shapiro–Wilk test (*p* > 0.05).

	TPC	DPPH	ABTS	FRAP	Protein
Time (min)	*p* < 0.05	*p* > 0.05	*p* < 0.05	*p* < 0.001	*p* > 0.05
10	25.62 ± 0.47	38.76 ± 0.52	57.21 ± 1.25	60.49 ± 0.83	18.04 ± 0.20
20	27.15 ± 0.48	39.55 ± 0.53	61.58 ± 1.26	66.19 ± 0.84	17.69 ± 0.20
Temperature (°C)	*p* < 0.05	*p* > 0.05	*p* < 0.05	*p* > 0.05	*p* > 0.05
130	25.70 ± 0.48	38.71 ± 0.53	57.60 ± 1.27	63.89 ± 0.84	18.07 ± 0.20
150	27.06 ± 0.47	39.59 ± 0.52	61.18 ± 1.24	62.79 ± 0.83	17.66 ± 0.20
Algae	*p* < 0.001	*p* < 0.001	*p* < 0.001	*p* < 0.001	*p* < 0.001
*L. hyperborea*	14.94 ± 0.48	31.91 ± 0.53	31.98 ± 1.26	58.58 ± 0.84	16.80 ± 0.20
*A. nodosum*	37.82 ± 0.47	46.39 ± 0.52	86.80 ± 1.24	68.10 ± 0.83	18.93 ± 0.20
Solvent	*p* < 0.001	*p* > 0.05	*p* < 0.001	*p* < 0.001	*p* < 0.001
AcNa/AL	22.40 ± 0.47	38.97 ± 0.52	63.19 ± 1.25	72.37 ± 0.83	16.62 ± 0.20
ChCl/AL	30.36 ± 0.48	39.34 ± 0.53	55.59 ± 1.26	54.31 ± 0.84	19.11 ± 0.20

Notes: A.n.—*A. nodosum*; L.h.—*L. hyperborea*. Solvents—choline chloride/lactic acid (ChCl/LA) and sodium acetate/lactic acid (AcNa/LA). Units of measurement: TPC—milligrams of gallic acid equivalents per gram of dry weight (mg GAE/g DW); DPPH, ABTS, and ORAC—Trolox equivalents (TE) in milligrams per gram of dry weight (mg TE/g DW); FRAP—g of ferrous equivalents (FE) per 100 g of dry weight (g FE/100 g DW); protein—g of bovine serum albumin equivalents (BSA) per of dry weight (g BSA/g DW). Different letters in columns in superscript are indicative of significant differences after the LSD post hoc test (*p* < 0.05).

**Table A4:** Mean results ± standard error (first-order interactions) of the optimized extraction contents for total phenolic content (TPC), antioxidant activity (2,2-Diphenyl-1-picrylhydrazyl (DPPH) radical scavenging activity, 2,2′-azino-bis (3-ethylbenzothiazoline-6-sulfonic acid) (ABTS) radical cation decolorization assay, ferric reducing antioxidant power (FRAP) assay), and protein. The significant differences were tested with full factorial ANOVAs. The homogeneity of variances was confirmed with Levene’s test (*p* > 0.05). The normal distribution of the residuals was confirmed with the Shapiro–Wilk test (*p* > 0.05).

	TPC	DPPH	ABTS	FRAP	Protein
Algae × Solvent	*p* < 0.001	*p* < 0.001	*p* < 0.05	*p* > 0.05	*p* < 0.001
A.n. × ChCl/AL	43.93 ± 0.67 ^d^	44.70 ± 0.74 ^c^	83.06 ± 1.77 ^c^	59.57 ± 1.18	21.83 ± 0.29 ^d^
A.n. × AcNa/AL	31.71± 0.67 ^c^	48.07 ± 0.74 ^d^	90.54 ± 1.77 ^d^	76.63 ± 1.18	16.02 ± 0.29 ^a^
L.h. × ChCl/AL	16.80 ± 0.67 ^b^	33.97 ± 0.75 ^b^	28.12 ± 1.79 ^a^	49.06 ± 1.19	16.38 ± 0.29 ^b^
L.h. × AcNa/AL	13.09 ± 0.67 ^a^	29.86 ± 0.74 ^a^	35.85± 1.77 ^b^	59.57 ± 1.18	17.22 ± 0.29 ^c^
Algae × Time	*p* < 0.05	*p* < 0.01	*p* < 0.001	*p* < 0.001	*p* < 0.01
A.n. × 10	36.27 ± 0.67 ^b^	44.86 ± 0.74 ^b^	87.69 ± 1.77 ^c^	58.88 ± 1.18 ^b^	18.64± 0.29 ^c^
A.n. × 20	39.37 ± 0.67 ^c^	47.92 ± 0.74 ^b^	85.91 ± 1.77 ^c^	77.32 ± 1.18 ^c^	19.21 ± 0.29 ^c^
L.h. × 10	14.96 ± 0.67 ^a^	32.65 ± 0.74 ^a^	26.72 ± 1.77 ^a^	62.09 ± 1.18 ^b^	17.44 ± 0.29 ^b^
L.h. × 20	14.92 ± 0.67 ^a^	31.18 ± 0.75 ^a^	37.24 ± 1.79 ^b^	55.07 ± 1.19 ^a^	16.16 ± 0.29 ^a^
Algae × Temperature	*p* > 0.05	*p* < 0.05	*p* > 0.05	*p* < 0.001	*p* > 0.05
A.n. × 130	37.25 ± 0.67 ^b^	46.86 ± 0.74 ^c^	86.68 ± 1.77 ^c^	74.62 ± 1.18 ^c^	19.33 ± 0.29
A.n. × 150	38.39 ± 0.67 ^b^	45.92 ± 0.74 ^c^	86.92 ± 1.77 ^c^	61.58 ± 1.18 ^b^	18.53 ± 0.29
L.h. × 130	14.15 ± 0.69 ^a^	30.57 ± 0.76 ^a^	28.53 ± 1.82 ^a^	53.16 ± 1.21 ^a^	16.81 ± 0.29
L.h. × 150	15.73 ± 0.66 ^a^	33.26 ± 0.73 ^b^	35.43 ± 1.74 ^b^	64.00 ± 1.16 ^b^	16.79 ± 0.28
Solvent × Time	*p* < 0.001	*p* > 0.05	*p* < 0.001	*p* > 0.05	*p* > 0.05
ChCl/AL × 10	28.35 ± 0.67 ^c^	38.33 ± 0.74	49.16 ± 1.77 ^a^	52.27 ± 1.18 ^a^	19.14 ± 0.29
ChCl/AL × 20	32.37 ± 0.67 ^d^	40.35 ± 0.75	62.02 ± 1.79 ^b^	56.36 ± 1.19 ^b^	19.08 ± 0.29
AcNa/AL × 10	22.88 ± 0.67 ^a^	39.18 ± 0.74	65.26 ± 1.77 ^b^	68.71 ± 1.18 ^c^	16.95 ± 0.29
AcNa/AL × 20	21.92± 0.67 ^b^	38.75 ± 0.74	61.13 ± 1.77 ^b^	76.03 ± 1.18 ^d^	16.30 ± 0.29
Solvent × Temperature	*p* < 0.001	*p* < 0.01	*p* < 0.001	*p* < 0.001	*p* < 0.001
ChCl/AL × 130	28.18 ± 0.69 ^b^	40.05 ± 0.76 ^b^	49.90 ± 1.82 ^a^	51.79 ± 1.21 ^a^	19.82 ± 0.29 ^c^
ChCl/AL × 150	32.64 ± 0.66 ^c^	38.63 ± 0.73 ^b^	61.28 ± 1.74 ^b^	56.84 ± 1.16 ^b^	18.40 ± 0.28 ^b^
AcNa/AL × 130	23.22 ± 0.69 ^a^	37.38 ± 0.74 ^a^	65.31 ± 1.77 ^b^	75.99 ± 1.18 ^d^	16.33 ± 0.29 ^a^
AcNa/AL × 150	24.41 ± 0.69 ^a^	40.55 ± 0.74 ^b^	61.08 ± 1.77 ^b^	68.75 ± 1.18 ^c^	16.91 ± 0.29 ^a^
Time × Temperature	*p* < 0.001	*p* > 0.05	*p* > 0.05	*p* < 0.001	*p* > 0.05
10 × 130	23.16 ± 0.67 ^a^	37.63 ± 0.74	54.16 ± 1.77 ^a^	38.76 ± 1.18 ^a^	18.22 ± 0.29
10 × 150	28.07 ± 0.67 ^b^	39.88 ± 0.74	60.25 ± 1.77 ^b^	82.22 ± 1.18 ^c^	17.87 ± 0.29
20 × 130	28.25 ± 0.69 ^c^	39.80 ± 0.76	61.05 ± 1.82 ^b^	89.02 ± 1.21 ^d^	17.93 ± 0.29
20 × 150	26.05 ± 0.66 ^d^	39.30 ± 0.73	62.11 ± 1.74 ^b^	43.37 ± 1.16 ^b^	17.44 ± 0.28

Notes: A.n.—*A. nodosum*; L.h.—*L. hyperborea*. Solvents—choline chloride/lactic acid (ChCl/LA) and sodium acetate/lactic acid (AcNa/LA). Units of measurement: TPC—milligrams of gallic acid equivalents per gram of dry weight (mg GAE/g DW); DPPH and ABTS—Trolox equivalents (TE) in milligrams per gram of dry weight (mg TE/g DW); FRAP—g of ferrous equivalents (FE) per 100 g of dry weight (g FE/100 g DW); protein—g of bovine serum albumin equivalents (BSA) per of dry weight (g BSA/g DW). Different letters in columns in superscript are indicative of significant differences after the LSD post hoc test (*p* < 0.05).

**Table A5:** Mean results ± standard error (second-order interactions) of the optimized extraction contents for total phenolic content (TPC), antioxidant activity (2,2-Diphenyl-1-picrylhydrazyl (DPPH) radical scavenging activity, 2,2′-azino-bis (3-ethylbenzothiazoline-6-sulfonic acid) (ABTS) radical cation decolorization assay, ferric reducing antioxidant power (FRAP) assay), and protein. The significant differences were tested with full factorial ANOVAs. The homogeneity of variances was confirmed with Levene’s test (*p* > 0.05). The normal distribution of the residuals was confirmed with the Shapiro–Wilk test (*p* > 0.05).

	TPC	DPPH	ABTS	FRAP	Protein
Algae × Solvent × Time	*p* < 0.001	*p* < 0.001	*p* < 0.01	*p* < 0.001	*p* < 0.01
A.n. × ChCl/AL × 10	39.60 ± 0.95 ^g^	44.07 ± 1.05 ^d^	82.64 ± 2.51 ^c^	57.44 ± 1.67 ^b^	21.84 ± 0.40 ^d^
A.n. × ChCl/AL × 20	48.26 ± 0.95 ^h^	45.34 ± 1.06 ^d^	83.48 ± 2.54 ^c^	61.71 ± 1.67 ^b^	21.82 ± 0.40 ^d^
A.n. × AcNa/AL × 10	32.94 ± 0.95 ^f^	45.65 ± 1.05 ^d^	92.74 ± 2.51 ^d^	60.33 ± 1.67 ^b^	15.45 ± 0.40 ^a^
A.n. × AcNa/AL × 20	30.49 ± 0.95 ^e^	50.50 ± 1.05 ^e^	88.34 ± 2.51 ^c^	92.9 3 ± 1.67 ^d^	16.60 ± 0.40 ^b^
L.h. × ChCl/AL × 10	17.10 ± 0.95 ^d^	32.59 ± 1.05 ^b^	15.67 ± 2.51 ^a^	47.10 ± 1.67 ^a^	16.44 ± 0.40 ^b^
L.h. × ChCl/AL × 20	16.49 ± 0.96 ^c^	35.36 ± 1.05 ^c^	40.56 ± 2.51 ^b^	51.01 ± 1.69 ^a^	16.33 ± 0.41 ^b^
L.h. × AcNa/AL × 10	12.83 ± 0.95 ^a^	32.72 ± 1.05 ^b^	37.77 ± 2.51 ^b^	77.08 ± 1.67 ^c^	18.44 ± 0.40 ^c^
L.h. × AcNa/AL × 20	13.35 ± 0.95 ^b^	26.99 ± 1.05 ^a^	33.92 ± 2.51 ^b^	59.12 ± 1.67 ^b^	15.99 ± 0.40 ^b^
Algae × Solvent × Temperature	*p* < 0.05	*p* > 0.05	*p* > 0.05	*p* < 0.001	*p* < 0.001
A.n. × ChCl/AL × 130	41.16 ± 0.95 ^f^	46.63 ± 1.10	79.18 ± 2.51	56.31 ± 1.67 ^a^	23.47 ± 0.40 ^d^
A.n. × ChCl/AL × 150	46.70 ± 0.95 ^g^	42.78 ± 1.01	86.94 ± 2.51	62.84 ± 1.67 ^b^	20.20 ± 0.40 ^c^
A.n. × AcNa/AL × 130	33.35 ± 0.95 ^e^	47.08 ± 1.05	94.18 ± 2.51	92.93 ± 1.67 ^d^	15.19 ± 0.40 ^a^
A.n. × AcNa/AL × 150	30.08 ± 0.95 ^d^	49.07 ± 1.05	86.90 ± 2.51	60.33 ± 1.67 ^b^	16.86 ± 0.40 ^b^
L.h. × ChCl/AL × 130	15.21 ± 0.99 ^b^	33.46 ± 1.05	20.62 ± 2.63	47.28 ± 1.75 ^a^	16.16 ± 0.42 ^b^
L.h. × ChCl/AL × 150	18.38 ± 0.91 ^c^	34.49 ± 1.05	35.61 ± 2.42	50.84 ± 1.61 ^a^	16.60 ± 0.39 ^b^
L.h. × AcNa/AL × 130	13.09 ± 0.95 ^a^	27.67 ± 1.05	36.43 ± 2.51	59.04 ± 1.67 ^b^	17.46 ± 0.40 ^b^
L.h. × AcNa/AL × 150	13.09 ± 0.95 ^a^	32.04 ± 1.05	35.26 ± 2.51	77.17 ± 1.67 ^c^	16.97 ± 0.40 ^b^
Algae × Time × Temperature	*p* < 0.001	*p* < 0.01	*p* > 0.05	*p* < 0.001	*p* > 0.05
A.n. × 10 × 130	32.50 ± 0.95 ^b^	45.78 ± 1.05 ^d^	84.91 ± 2.51	32.62 ± 1.67 ^a^	18.80 ± 0.40
A.n. × 10 × 150	40.04 ± 0.95 ^d^	43.94 ± 1.05 ^c^	90.47 ± 2.51	85.14 ± 1.67 ^g^	18.49 ± 0.40
A.n. × 20 × 130	42.01 ± 0.95 ^e^	47.94 ± 1.05 ^d^	88.45 ± 2.51	116.62 ± 1.67 ^h^	19.86 ± 0.40
A.n. × 20 × 150	36.73 ± 0.95 ^c^	47.91 ± 1.05 ^d^	83.37 ± 2.51	38.02 ± 1.67 ^b^	18.56 ± 0.40
L.h. × 10 × 130	13.82 ± 0.95 ^a^	29.48 ± 1.05 ^a^	23.41 ± 2.51	44.89 ± 1.67 ^c^	17.63 ± 0.40
L.h. × 10 × 150	16.11 ± 0.95 ^a^	35.83 ± 1.05 ^b^	30.03 ± 2.51	79.29 ± 1.67 ^f^	17.25 ± 0.40
L.h. × 20 × 130	14.48 ± 0.99 ^a^	31.66 ± 1.10 ^a^	33.64 ± 2.63	61.42 ± 1.75 ^e^	16.00 ± 0.42
L.h. × 20 × 150	15.36 ± 0.91 ^a^	30.70 ± 1.01 ^a^	40.84 ± 2.42	48.71 ± 1.61 ^d^	16.32 ± 0.39
Solvent × Time × Temperature	*p* < 0.01	*p* > 0.05	*p* > 0.05	*p* < 0.001	*p* > 0.05
ChCl/AL × 10 × 130	23.39 ± 0.95 ^b^	38.35 ± 1.05	43.76 ± 2.51	35.59 ± 1.67 ^a^	19.97 ± 0.40
ChCl/AL × 10 × 150	33.14 ± 0.95 ^c^	38.31 ± 1.05	54.55 ± 2.51	68.95 ± 1.67 ^c^	18.31 ± 0.40
ChCl/AL × 20 × 130	32.98 ± 0.99 ^c^	41.75 ± 1.10	56.04 ± 2.63	67.99 ± 1.75 ^c^	19.67 ± 0.42
ChCl/AL × 20 × 150	31.77 ± 0.91 ^c^	38.95 ± 1.01	68.00 ± 2.42	44.73 ± 1.61 ^b^	18.49 ± 0.39
AcNa/AL × 10 × 130	22.93 ± 0.95 ^b^	36.91 ± 1.05	64.56 ± 2.51	41.92 ± 1.67 ^b^	16.46 ± 0.40
AcNa/AL × 10 × 150	22.83 ± 0.95 ^b^	41.46 ± 1.05	65.95 ± 2.51	95.49 ± 1.67 ^d^	17.43 ± 0.40
AcNa/AL × 20 × 130	23.51 ± 0.95 ^b^	37.85 ± 1.05 v	66.06 ± 2.51	110.05 ± 1.67 ^e^	16.19 ± 0.40
AcNa/AL × 20 × 150	20.33 ± 0.95 ^a^	39.65 ± 1.05	56.21 ± 2.51	42.01 ± 1.67 ^b^	16.40 ± 0.40

Notes: A.n.—*A. nodosum*; L.h.—*L. hyperborea*. Solvents—choline chloride/lactic acid (ChCl/LA) and sodium acetate/lactic acid (AcNa/LA). Units of measurement: TPC—milligrams of gallic acid equivalents per gram of dry weight (mg GAE/g DW); DPPH and ABTS—Trolox equivalents (TE) in milligrams per gram of dry weight (mg TE/g DW); FRAP—g of ferrous equivalents (FE) per 100 g of dry weight (g FE/100 g DW); protein—g of bovine serum albumin equivalents (BSA) per of dry weight (g BSA/g DW). Different letters in columns in superscript are indicative of significant differences after the LSD post hoc test (*p* < 0.05).

### Appendix A.3. Complementary Tables for Section 3.4.4 Efficiency of Bioactive Compound Extraction Using Microwave-Assisted Extraction and DESs

**Table A6:** Mean results ± standard error (factors) of the optimized extraction contents of the different phenolic compounds: 3,4-dihydroxybenzoic acid (DHB), phthalic acid (PHT), salicylic acid (SAL), 4-hydroxybenzoic acid (HBA), and vanillin (VAN). The significant differences were tested with full factorial ANOVAs. The homogeneity of variances was confirmed with Levene’s test (*p* > 0.05). The normal distribution of the residuals was confirmed with the Shapiro–Wilk test (*p* > 0.05).

	DHB	PHT	SAL	HBA	VAN
TT	*p* < 0.001	*p* < 0.001	*p* > 0.05	*p* < 0.001	*p* < 0.001
10,150	310.92 ± 3.7	37.46 ± 2.99	2.18 ± 0.15	7.32 ± 0.10	2740.39 ± 29.27
20,130	17.03 ± 3.7	60.91 ± 2.99	1.87 ± 0.15	7.94 ± 0.10	468.95 ± 29.27
Algae	*p* < 0.001	*p* < 0.001	*p* < 0.001	*p* < 0.05	*p* < 0.001
*L. hyperborea*	136.55 ± 3.7	59.57 ± 2.99	1.35 ± 0.15	7.45 ± 0.10	1994.59 ± 29.27
*A. nodosum*	191.40 ± 3.7	38.80 ± 2.99	2.70 ± 0.15	7.81 ± 0.10	1214.75 ± 29.27
Solvent	*p* < 0.001	*p* < 0.001	*p* < 0.001	*p* < 0.001	*p* < 0.001
AcNa/LA	310.89 ± 3.7	40.31 ± 2.99	2.76 ± 0.15	8.4 ± 0.10	3106.13 ± 29.27
ChCl/LA	17.05 ± 3.7	58.06 ± 2.99	1.29 ± 0.15	6.83 ± 0.10	103.22 ± 29.27

Notes: TT—time/temperature combination (min, °C). A.n.—*A. nodosum*; L.h.—*L. hyperborea*. Solvents—choline chloride/lactic acid (ChCl/LA) and sodium acetate/lactic acid (AcNa/LA). Unit of measurement: µg/g dry weight (µg/g DW). Different letters in columns in superscript are indicative of significant differences after the LSD post hoc test (*p* < 0.05).

**Table A7:** Mean results ± standard error (first-order interaction) of the optimized extraction contents of the different phenolic compounds: 3,4-dihydroxybenzoic acid (DHB), phthalic acid (PHT), salicylic acid (SAL), 4-hydroxybenzoic acid (HBA), and vanillin (VAN). The significant differences were tested with full factorial ANOVAs. The homogeneity of variances was confirmed with Levene’s test (*p* > 0.05). The normal distribution of the residuals was confirmed with the Shapiro–Wilk test (*p* > 0.05).

	DHB	PHT	SAL	HBA	VAN
Algae × Solvent	*p* < 0.001	*p* < 0.001	*p* < 0.05	*p* > 0.05	*p* < 0.001
A.n. × ChCl/LA	28.65 ± 5.2 ^b^	33.70 ± 4.23 ^a^	2.28 ± 0.21 ^b^	6.89 ± 0.14	132.25 ± 41.39 ^a^
A.n. × AcNa/LA	354.15 ± 5.2 ^d^	43.90 ± 4.23 ^b^	3.12 ± 0.21 ^d^	8.73 ± 0.14	2297.25 ± 41.39 ^b^
L.h. × ChCl/LA	5.46 ± 5.2 ^a^	82.42 ± 4.23 ^c^	0.31 ± 0.21 ^a^	6.76 ± 0.14	74.19 ± 41.39 ^a^
L.h. × AcNa/LA	267.63 ± 5.2 ^c^	36.71 ± 4.23 ^b^	2.40 ± 0.21 ^c^	8.14 ± 0.14	3915.00 ± 41.39 ^c^
Algae × TT	*p* < 0.001	*p* < 0.05	*p* > 0.05	*p* > 0.05	*p* < 0.001
A.n. × 20,130	18.41 ± 5.2 ^b^	45.25 ± 4.23 ^b^	2.52 ± 0.21	8.19 ± 0.14	348.87 ± 41.39 ^a^
A.n. × 10,150	364.38 ± 5.2 ^c^	32.35 ± 4.23 ^a^	2.88 ± 0.21	7.43 ± 0.14	2080.63 ± 41.39 ^c^
L.h. × 20,130	15.64 ± 5.2 ^a^	76.57 ± 4.23 ^c^	1.23 ± 0.21	7.69 ± 0.14	589.03 ± 41.39 ^b^
L.h. × 10,150	257.45 ± 5.2 ^c^	42.57 ± 4.23 ^a^	1.48 ± 0.21	7.22 ± 0.14	3400.16 ± 41.39 ^d^
Solvent × TT	*p* < 0.001	*p* < 0.001	*p* > 0.05	*p* < 0.001	*p* < 0.001
ChCl/LA × 20,130	3.85 ± 5.2 ^a^	80.21 ± 4.23 ^b^	1.29 ± 0.21	7.49 ± 0.14 ^b^	75.40 ± 41.39 ^a^
ChCl/LA × 10,150	30.26 ± 5.2 ^b^	35.92 ± 4.23 ^a^	1.30 ± 0.21	6.16 ± 0.14 ^a^	131.03 ± 41.39 ^a^
AcNa/LA × 20,130	30.21 ± 5.2 ^b^	41.61 ± 4.23 ^a^	2.46 ± 0.21	8.39 ± 0.14 ^c^	862.50 ± 41.39 ^b^
AcNa/LA × 10,150	591.58 ± 5.2 ^c^	39.00 ± 4.23 ^a^	3.06 ± 0.21	8.49 ± 0.14 ^c^	5349.75 ± 41.39 ^c^

Notes: TT—time/temperature combination (min, °C). A.n.—*A. nodosum*; L.h.—*L. hyperborea*. Solvents—choline chloride/lactic acid (ChCl/LA) and sodium acetate/lactic acid (AcNa/LA). Unit of measurement: µg/g dry weight (µg/g DW). Different letters in columns in superscript are indicative of significant differences after the LSD post hoc test (*p* < 0.05).

### Appendix A.4. Complementary Tables for Section 3.6 Characterization of Residues Resulting from the Extraction of Bioactive Compounds

**Table A8:** Mean results ± standard error (factors) of the optimized extraction residue polysaccharides glucan (GLU), xylan/galactan/mannan (XGM), fucoidan (FUC), and acid-insoluble residue (AIR). The significant differences were tested with full factorial ANOVAs. The homogeneity of variances was confirmed with Levene’s test (*p* > 0.05). The normal distribution of the residuals was confirmed with the Shapiro–Wilk test (*p* > 0.05).

	GLU	XGM	FUC	AIR
TT	*p* < 0.05	*p* < 0.05	*p* < 0.001	*p* < 0.001
10,150	13.72 ± 0.28	3.42 ± 0.10	1.01 ± 0.12	45.41 ± 0.15
20,130	13.39 ± 0.28	3.05 ± 0.10	1.77 ± 0.12	27.99 ± 0.15
Algae	*p* < 0.001	*p* < 0.001	*p* > 0.05	*p* < 0.001
*L. hyperborea*	17.47 ± 0.28	3.71 ± 0.10	1.35 ± 0.12	18.78 ± 0.15
*A. nodosum*	9.64 ± 0.28	2.75 ± 0.10	1.43 ± 0.12	54.61 ± 0.15
Solvent	*p* < 0.001	*p* > 0.05	*p* < 0.001	*p* < 0.001
AcNa/AL	12.03 ± 0.28	3.21 ± 0.10	0.75 ± 0.12	38.70 ± 0.15
ChCl/AL	15.09 ± 0.28	3.25 ± 0.10	2.04 ± 0.12	34.70 ± 0.15

Notes: TT—time/temperature combination (min, °C). Solvents—choline chloride/lactic acid (ChCl/LA) and sodium acetate/lactic acid (AcNa/LA). Unit of measurement: g/100 g or percentage.

**Table A9:** Mean results ± standard error (first-order interaction) of the optimized extraction residue polysaccharides glucan (GLU), xylan/galactan/mannan (XGM), fucoidan (FUC), and acid-insoluble residue (AIR). The differences were tested with full factorial ANOVAs. Homogeneity of variances was confirmed via Levene’s test (*p* > 0.05). The normal distribution of residuals was confirmed via the Shapiro–Wilk test (*p* > 0.05).

	GLU	XGM	FUC	AIR
Algae × Solvent	*p* < 0.001	*p* < 0.001	*p* > 0.05	*p* < 0.001
A.n. × ChCl/AL	10.16 ± 0.40 ^a^	3.29 ± 0.14 ^b^	2.16 ± 0.17	47.10 ± 0.21 ^c^
A.n. × AcNa/AL	9.13 ± 0.40 ^a^	2.21 ± 0.14 ^a^	0.70 ± 0.17	62.13 ± 0.21 ^d^
L.h. × ChCl/AL	20.02 ± 0.40 ^c^	3.21 ± 0.14 ^b^	1.91 ± 0.17	22.29 ± 0.21 ^b^
L.h. × AcNa/AL	14.93 ± 0.40 ^b^	4.21 ± 0.14 ^c^	0.79 ± 0.17	15.28 ± 0.21 ^a^
Algae × TT	*p* > 0.05	*p* < 0.001	*p* > 0.05	*p* < 0.001
A.n. × 20,130	9.18 ± 0.40	3.08 ± 0.14 ^b^	1.63 ± 0.17	37.63 ± 0.21
A.n. × 10,150	10.10 ± 0.40	2.42 ± 0.14 ^a^	1.23 ± 0.17	71.60 ± 0.21
L.h. × 20,130	17.60 ± 0.40	3.01 ± 0.14 ^b^	1.91 ± 0.17	18.34 ± 0.21
L.h. × 10,150	17.34 ± 0.40	4.41 ± 0.14 ^c^	0.79 ± 0.17	19.23 ± 0.21
Solvent × TT	*p* < 0.01	*p* > 0.05	*p* < 0.001	*p* > 0.05
ChCl/AL × 20,130	14.15 ± 0.40 ^c^	3.13 ± 0.14	2.05 ± 0.17 ^c^	26.02 ± 0.21 ^a^
ChCl/AL × 10,150	16.03 ± 0.40 ^d^	3.38 ± 0.14	2.02 ± 0.17 ^c^	43.38 ± 0.21 ^c^
AcNa/AL × 20,130	12.64 ± 0.40 ^b^	2.97 ± 0.14	1.49 ± 0.17 ^b^	29.95 ± 0.21 ^b^
AcNa/AL × 10,150	11.41 ± 0.40 ^a^	3.46 ± 0.14	≈0 ^a^	47.45 ± 0.21 ^d^

Notes: TT—time/temperature combination (min, °C). Solvents—choline chloride/lactic acid (ChCl/LA) and sodium acetate/lactic acid (AcNa/LA). Unit of measurement: g/100 g or percentage. Different letters in columns in superscript are indicative of significant differences after the LSD post hoc test (*p* < 0.05).
